# Characterization of *Acinetobacter baumannii* Copper Resistance Reveals a Role in Virulence

**DOI:** 10.3389/fmicb.2020.00016

**Published:** 2020-02-06

**Authors:** Caitlin L. Williams, Heather M. Neu, Yonas A. Alamneh, Ryan M. Reddinger, Anna C. Jacobs, Shweta Singh, Rania Abu-Taleb, Sarah L. J. Michel, Daniel V. Zurawski, D. Scott Merrell

**Affiliations:** ^1^Department of Microbiology & Immunology, Uniformed Services University, Bethesda, MD, United States; ^2^Department of Pharmaceutical Sciences, School of Pharmacy, University of Maryland, Baltimore, Baltimore, MD, United States; ^3^Wound Infections Department, Bacterial Diseases Branch, Walter Reed Army Institute of Research, Silver Spring, MD, United States

**Keywords:** *Acinetobacter baumannii*, metal, copper, pathogenesis, *Galleria*

## Abstract

*Acinetobacter baumannii* is often highly drug-resistant and causes severe infections in compromised patients. These infections can be life threatening due to limited treatment options. Copper is inherently antimicrobial and increasing evidence indicates that copper containing formulations may serve as non-traditional therapeutics against multidrug-resistant bacteria. We previously reported that *A. baumannii* is sensitive to high concentrations of copper. To understand *A. baumannii* copper resistance at the molecular level, herein we identified putative copper resistance components and characterized 21 strains bearing mutations in these genes. Eight of the strains displayed a copper sensitive phenotype (*pcoA*, *pcoB*, *copB, copA/cueO, copR/cusR, copS/cusS, copC, copD*); the putative functions of these proteins include copper transport, oxidation, sequestration, and regulation. Importantly, many of these mutant strains still showed increased sensitivity to copper while in a biofilm. Inductively coupled plasma mass spectrometry revealed that many of these strains had defects in copper mobilization, as the mutant strains accumulated more intracellular copper than the wild-type strain. Given the crucial antimicrobial role of copper-mediated killing employed by the immune system, virulence of these mutant strains was investigated in *Galleria mellonella*; many of the mutant strains were attenuated. Finally, the *cusR* and *copD* strains were also investigated in the murine pneumonia model; both were found to be important for full virulence. Thus, copper possesses antimicrobial activity against multidrug-resistant *A. baumannii*, and copper sensitivity is further increased when copper homeostasis mechanisms are interrupted. Importantly, these proteins are crucial for full virulence of *A. baumannii* and may represent novel drug targets.

## Introduction

*Acinetobacter baumannii* is responsible for a significant proportion of nosocomial infections worldwide, and an even greater number of ICU-acquired infections; co-morbidities are an important risk factor for *A. baumannii* infection (Vincent et al., 2009; Lob et al., 2016; Wong et al., 2017; Du et al., 2019). Associated types of infections are diverse, and include pneumonia, urinary tract infections, bacteremia, skin and soft tissue infections, osteomyelitis, and meningitis (Peleg et al., 2008; Wong et al., 2017). Extensive drug-resistance is common among *A. baumannii* strains, and resistance limits treatment options and leads to higher morbidity and mortality (Antunes et al., 2014; Giammanco et al., 2017; Du et al., 2019). Indeed, multidrug-resistant *A. baumannii* has been named a “serious” threat by the CDC since 2013, and in 2017 carbapenem-resistant *A. baumannii* topped the World Health Organization’s Priority Pathogen’s List as a “Level 1: Critical priority” pathogen (Centers for Disease Control and Prevention, 2013; World Health Organization, 2017). Clearly, new therapeutic options are desperately needed to treat multidrug-resistant *A. baumannii* infections.

Antibiotics target essential functions for bacterial growth and/or survival. Metal homeostasis is a vital process that provides an extensive list of potential new antimicrobial targets. Copper is required for cellular function, e.g., for redox balance and as an enzyme cofactor. However, copper ions become toxic at high concentrations; thus, it is important that intracellular copper levels be tightly controlled. Copper ions cause damage by participating in Fenton-like chemistry to produce hydroxyl radicals that react with and damage essential biomolecules (Liochev and Fridovich, 2002) and also by displacing iron from crucial iron-sulfur cluster proteins (Macomber and Imlay, 2009). Studies in *Escherichia coli* and *Salmonella* spp. have shown that when bacteria are placed on copper surfaces, outer membrane integrity is compromised, hydroxyl radicals are produced, respiration is inhibited, and DNA is degraded (Warnes et al., 2012). Fenton-like chemistry-based killing of pathogens also occurs in the host via host-generated reactive oxygen species. Because of the need for new therapeutics to treat antibiotic resistant pathogens, research into the use of copper as an antimicrobial has lately increased. For example, the use of copper-containing surfaces in hospitals has been shown to greatly reduce environmental contamination with nosocomial pathogens and to reduce rates of health care-acquired infections (Salgado et al., 2013; Sifri et al., 2016; von Dessauer et al., 2016). Additionally, copper-containing wound dressings are in development to aid in healing of infected wounds (Borkow et al., 2010; Ahire et al., 2016).

The damaging effects of copper have been harnessed by the host immune system. Indeed, phagocytic immune cells employ a copper burst within the phagosome to kill pathogens (Sheldon and Skaar, 2019). Concentrations of copper upwards of 0.5 mM have been measured in macrophage phagosomes (Wagner et al., 2005). Additionally, host mobilization of copper also occurs in response to infection: increased concentrations of copper have been measured in serum and in wound exudate (Milanino et al., 1993; Jones et al., 2001). Consequently, pathogens with mutations in crucial copper resistance genes have been found to have impaired intracellular survival, colonization, and/or virulence (Djoko et al., 2015). Furthermore, it was recently shown that a copper sensitive mutant strain of *A. baumannii* demonstrated reduced colonization of the respiratory tract of mice (Alquethamy et al., 2019).

**FIGURE 1:**
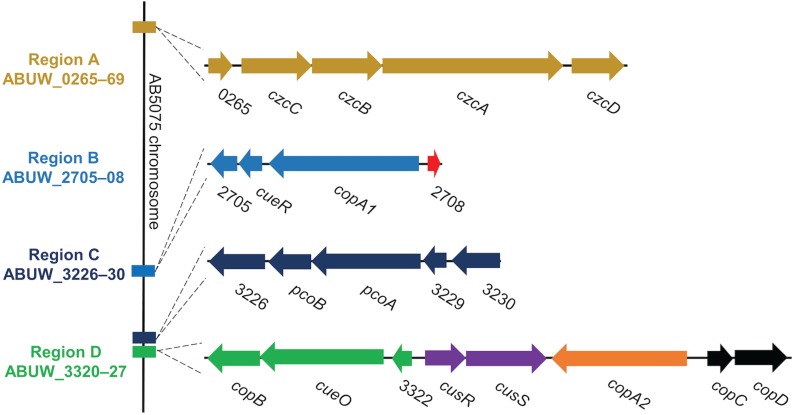
Organization of regions A–D in the AB5075 chromosome. We previously identified 22 putative copper-related genes in the genome of AB5075 (Williams et al., 2016). These genes are located in four chromosomal regions named A–D. The relative location of each region on the AB5075 chromosome is shown on the left (not to scale), and the genes and regions are depicted to scale relative to one another. Each operon is colored uniquely, and the same colors are maintained in Figure 9. Gene annotations are shown when available, otherwise ORF numbers are used.

In bacterial species where copper homeostasis has been well-characterized, a variety of proteins are utilized to facilitate copper homeostasis. Though this process remains poorly understood in *A. baumannii*, recent studies have identified many putative copper resistance gene homologs in *A. baumannii* clinical isolates (Hernandez-Montes et al., 2012; Williams et al., 2016; Alquethamy et al., 2019). For example, in the model clinical multidrug-resistant isolate, AB5075, copper resistance genes exist in four distinct chromosomal regions named A–D (Figure 1) (Williams et al., 2016). These regions contain genes predicted to encode homologs of the most important copper resistance proteins of bacteria, e.g., copper ATPases, copper oxidases, copper transporters, copper chaperones, and regulatory proteins (Williams et al., 2016).

Region A includes genes for a large RND family efflux pump, *czcCBAD*, and a hypothetical protein ABUW_0265, which is located at the start of the operon. A similar type of pump, CusCFBA, has been shown to contribute to copper resistance in *E. coli* (Franke et al., 2003). While the genes in this operon are annotated as copper-related in AB5075, expression does not increase in response to copper exposure (Williams et al., 2016) and it has been suggested that *A. baumannii* in fact does not encode *cus* homologs (Alquethamy et al., 2019), thus the role of this operon in copper resistance is very tentative.

Region B encodes four genes, including homologs of two known copper resistance proteins. CueR, CopA1, and the hypothetical protein ABUW_2705 are encoded as an operon; divergently transcribed from that operon is a putative copper chaperone, ABUW_2708, recently named CopZ (Alquethamy et al., 2019). CueR has been well-studied in *E. coli* and is a cytoplasmic transcription factor that binds copper ions with exquisite sensitivity (Changela et al., 2003). Upon copper binding, CueR positively regulates transcription of copper resistance genes. In *E. coli*, CueR is known to regulate the expression of copper ATPase CopA and the copper oxidase CueO (Outten et al., 2000; Yamamoto and Ishihama, 2005). The CueR regulon in *A. baumannii* is not yet characterized, however, a putative CueR binding site has been identified in the intergenic space of region B, suggesting that all of the genes in region B may be regulated by CueR (Alquethamy et al., 2019). Appropriately, the genes of region B are all upregulated in response to copper in a similar manner (Williams et al., 2016). A CopA1 homolog, also encoded in region B, is a putative copper ATPase, which is another important and well-characterized copper resistance protein. Copper ATPases use energy from ATP to move copper ions into the periplasmic space from the cytoplasm and are known to contribute to copper resistance in a number of bacteria (Rensing et al., 2000). Very recently, the copper ATPase in region B was demonstrated to contribute to copper resistance of *A. baumannii* AB5075 *in vitro* and to contribute to colonization in a mouse respiratory infection model (Alquethamy et al., 2019).

Region C encodes a single operon of five genes, ABUW_3226 – 3230. Two of the genes, ABUW_3227 and ABUW_3228, are annotated as *pcoB* and *pcoA*, respectively. In *E. coli*, the *pco* system is plasmid-based; while the Pco proteins seem to be homologous to known chromosomally encoded copper resistance factors, their functions are less well-studied and understood (Lee et al., 2002). In AB5075, PcoB is homologous to CopB, an outer membrane protein that may function as a copper transporter (Cha and Cooksey, 1993). PcoA is homologous to CueO, a periplasmic copper oxidase that detoxifies by oxidizing copper ions to their less damaging form (Grass and Rensing, 2001). Surprisingly, expression of the genes in region C does not change in response to copper exposure (Williams et al., 2016), however that does not preclude the possibility that these genes are indeed involved in copper resistance.

Region D is the largest of the identified regions in AB5075; it contains a total of eight genes arranged as two pairs of divergently transcribed genes/operons. Among these eight genes are seven homologs of characterized copper resistance proteins: CopB, CueO, CusR, CusS, CopA2, CopC, and CopD. The functions of homologs of some of these copper-related proteins have been previously elucidated in other bacteria. CopB and CopD are membrane proteins and putative copper transporters, though not yet well-characterized (Cha and Cooksey, 1993). CueO is a periplasmic copper oxidase, which detoxifies by oxidizing cuprous copper ions (Cu^1+^) to their less damaging cupric (Cu^2+^) form (Grass and Rensing, 2001). CopA2 is a second copper ATPase (the other being CopA1 of region B); previous work did not reveal a role for *copA2* in copper resistance in AB5075 (Alquethamy et al., 2019). CopC is a periplasmic copper chaperone protein that likely contributes to copper resistance by binding copper ions as a means to prevent reactivity and/or by shuttling ions between other copper resistance proteins (Puig et al., 2002; Arnesano et al., 2003; Padilla-Benavides et al., 2014). CusR and CusS form a two-component system that senses periplasmic copper ions and activates the CusR regulon in response (Gudipaty et al., 2012). In *E. coli*, CusR is known to directly regulate expression of the *cusRS* operon and the divergently transcribed *cusCFBA* operon (Munson et al., 2000; Yamamoto and Ishihama, 2005; Gudipaty et al., 2012). It is not yet known which genes comprise the CusR regulon in *A. baumannii*, however, putative CusR binding sites were identified in the intergenic regions upstream of the four operons in region D, suggesting that all of region D may be regulated by CusR (Alquethamy et al., 2019).

Notably, not all clinical isolates of *A. baumannii* carry all of the copper-related genes found in AB5075. Based on previous analyses of two panels of clinical isolates, as well as the common lab strain ATCC 17978, region A–C genes are encoded by all strains. Conversely, region D genes are found in about 40% of strains (5/12 strains) (Williams et al., 2016; Hassan et al., 2017). Furthermore, the presence of region D genes is linked to increased copper resistance. Indeed, isolates show marked differences in copper sensitivity that directly correlates with the presence of the region D genes; clinical isolates lacking region D demonstrate significantly less copper resistance than isolates that encode the region D genes (Williams et al., 2016). Moreover, expression of the genes in region D dramatically increases in response to copper exposure (Williams et al., 2016). These results strongly suggest that the region D genes are crucial for extensive copper homeostasis capability in *A. baumannii*.

Given the growing interest in targeting copper homeostasis as a novel antimicrobial strategy and the lack of understanding of which factors contribute to copper resistance in *A. baumannii*, we sought to identify genes that were important for copper resistance in this important pathogen. Herein, we describe a detailed analysis of 21 mutant strains, each bearing insertions in the genes carried in regions A–D (Figure 1). Marked copper sensitivity of a subset of the mutant strains as well as defects in virulence are described.

## Materials and Methods

### Bacterial Strains and Growth Conditions

Bacterial strains are listed in Table 1. Strains were routinely streaked from freezer stocks and were grown overnight at 37°C on lysogeny broth (LB) 1.5% agar plates (MoBio, Carlsbad, CA, United States). Liquid cultures of *A. baumannii* were grown at 37°C shaking at 190 rpm in LB or in M9 minimal medium (Sambrook and Russell, 2001) supplemented with 0.1% casamino acids (Difco, Franklin Lakes, NJ, United States); the M9 medium was always supplemented with amino acids, but for simplicity is referred to as ‘M9 medium’ throughout the manuscript. Importantly, overnight cultures were consistently started from a single opaque colony, as phase variation has been shown to affect growth and virulence (Tipton et al., 2015). Overnight growth was restricted to a period of 16–19 h. *A. baumannii* strains freezer stocks were maintained at −80°C in medium supplemented with 40% glycerol.

**TABLE 1:** Strains used in this study.

**Transposon mutant strains**						

**Strain name**	**Shorthand name^1^**	**Locus tag**	**Strain name in transposon library^2^**	**Tn*5* location in ORF^2^**	**Lab designation**	**Reference**
AB5075	Wild-type				DSM1866	Jacobs et al., 2014a
AB5075 *pcoB*140:T26	*pcoB*:T26	ABUW_3227	tnab1_kr121204p07q140	445 (756)	DSM1833	Gallagher et al., 2015
AB5075 *pcoA*104:T26	*pcoA*:T26	ABUW_3228	tnab1_kr121203p02q104	607 (1935)	DSM1823	Gallagher et al., 2015
AB5075 *copB*156:T26	*copB*:T26	ABUW_3320	tnab1_kr121203p05q156	142 (903)	DSM1837	Gallagher et al., 2015
AB5075 *copA*/*cueO*118:T26	*cueO*:T26	ABUW_3321	tnab1_kr121128p03q118	381 (2118)	DSM1840	Gallagher et al., 2015
AB5075 *copR*/*cusR*106:T26	*cusR*:T26	ABUW_3323	tnab1_kr121128p07q106	195 (684)	DSM1825	Gallagher et al., 2015
AB5075 *copS*/*cusS*128:T26	*cusS*:T26	ABUW_3324	tnab1_kr121205p01q128	328 (1377)	DSM1832	Gallagher et al., 2015
AB5075 *copC*160:T26	*copC*:T26	ABUW_3326	tnab1_kr121119p04q160	46 (381)	DSM1844	Gallagher et al., 2015
AB5075 *copD*117:T26	*copD*:T26	ABUW_3327	tnab1_kr121204p06q117	280 (882)	DSM1830	Gallagher et al., 2015
AB5075 ABUW_0265-193:T26		ABUW_0265	tnab1_kr130913p10q193	177 (399)	DSM1856	Gallagher et al., 2015
AB5075 *czcC*151:T26		ABUW_0266	tnab1_kr121127p01q151	525 (1311)	DSM1849	Gallagher et al., 2015
AB5075 *czcB*187:T26		ABUW_0267	tnab1_kr121205p01q187	70 (1218)	DSM1848	Gallagher et al., 2015
AB5075 *czcA*162:T26		ABUW_0268	tnab1_kr121204p04q162	1523 (3159)	DSM1846	Gallagher et al., 2015
AB5075 *czcD*174:T26		ABUW_0269	tnab1_kr121205p05q174	486 (957)	DSM1851	Gallagher et al., 2015
AB5075 ABUW_2705-139:T26		ABUW_2705	tnab1_kr130903p04q139	89 (408)	DSM1857	Gallagher et al., 2015
AB5075 *cueR*139:T101		ABUW_2706	tnab1_jr130919p01q139	238 (402)	DSM1858	Gallagher et al., 2015
AB5075 *actP2*/*copA1*-181:T26		ABUW_2707	tnab1_kr121128p08q181	791 (2472)	DSM1822	Gallagher et al., 2015
AB5075 ABUW_3226-176:T26		ABUW_3226	tnab1_kr121211p04q176	193 (996)	DSM1859	Gallagher et al., 2015
AB5075 ABUW_3229-182:T26		ABUW_3229	tnab1_kr130913p04q182	86 (399)	DSM1860	Gallagher et al., 2015
AB5075 ABUW_3230-184:T26		ABUW_3230	tnab1_kr130904p01q184	234 (822)	DSM1861	Gallagher et al., 2015
AB5075 ABUW_3322-131:T26		ABUW_3322	tnab1_kr130904p04q131	25 (366)	DSM1862	Gallagher et al., 2015
AB5075 *actP1*/*copA2*-159:T26		ABUW_3325	tnab1_kr121203p05q159	784 (2346)	DSM1828	Gallagher et al., 2015

**Complemented mutant strains^3^**						

**Proper name**	**Shorthand name^1^**			**Lab designation**		**Reference**

AB5075 *pcoB*140:T26 *att*Tn*7*-*hph*-*pcoB*	*pcoB*:T26^C^			DSM1904		This study
AB5075 *pcoA*104:T26 *att*Tn*7*-*hph*-*pcoA*	*pcoA*:T26^C^			DSM1905		This study
AB5075 *copB*156:T26 *att*Tn*7*-*hph*-*copB*	*copB*:T26^C^			DSM1901		This study
AB5075 *copA*/*cueO*118:T26 *att*Tn*7*-*hph*-*cueO*	*cueO*:T26^C^			DSM1906		This study
AB5075 *copR*/*cusR*106:T26 *att*Tn*7*-*hph*-*cusR*	*cusR*:T26^C^			DSM1890		This study
AB5075 *copS*/*cusS*128:T26 *att*Tn*7*-*hph*-*cusS*	*cusS*:T26^C^			DSM1902		This study
AB5075 *copC*160:T26 *att*Tn*7*-*hph*-*copC*	*copC*:Tn5^C^			DSM1897		This study
AB5075 *copD*117:T26 *att*Tn*7*-*hph*-*copD*	*copD*:Tn5^C^			DSM1898		This study

**Mating strains**						

**Strain name**	**Description**			**Lab designation**		**Reference**

HB101/pRK2013	Mating helper strain (*oriT* helper)			DSM1881		Kumar et al., 2010
DH5αλpir/pTNS2	Mating helper strain (transposase)			DSM1880		Kumar et al., 2010
DH5α/pUC18T mini-Tn*7*-*hph*	Tn*7* with hygromycin resistance gene, *hph*, and MCS			DSM1883		Jacobs et al., 2014a
DH5α/pUC18T mini-Tn*7*-*hph*-*pcoA*	Tn*7* with *hph* and *pcoA* with native promoter			DSM1894		This study
DH5α/pUC18T mini-Tn*7*-*hph*-*pcoB*	Tn*7* with *hph* and *pcoB* with native promoter			DSM1895		This study
DH5α/pUC18T mini-Tn*7*-*hph*-*copB*	Tn*7* with *hph* and *copB* with native promoter			DSM1899		This study
DH5α/pUC18T mini-Tn*7*-*hph*-*cueO*	Tn*7* with *hph* and *cueO* with native promoter			DSM1903		This study
DH5α/pUC18T mini-Tn*7*-*hph*-*cusR*	Tn*7* with *hph* and *cusR* with native promoter			DSM1889		This study
DH5α/pUC18T mini-Tn*7*-*hph*-*cusS*	Tn*7* with *hph* and *cusS* with native promoter			DSM1900		This study
DH5α/pUC18T mini-Tn*7*-*hph*-*copC*	Tn*7* with *hph* and *copC* with native promoter			DSM1893		This study
DH5α/pUC18T mini-Tn*7*-*hph*-*copD*	Tn*7* with *hph* and *copD* with native promoter			DSM1896		This study

**Additional strains**						

**Strain name**	**Description**			**Lab designation**		**Reference**

AB4857/pAJ100	AB4857 with empty vector, pAJ100			DSM1908		This study
AB4857/pAJ100 – Region D	AB4857 with pAJ100 carrying Region D			DSM1910		This study
AB5711/pAJ100	AB5711 with empty vector, pAJ100			DSM1909		This study
AB5711/pAJ100 – Region D	AB5711 with pAJ100 carrying Region D			DSM1911		This study
*E. coli*/pAJ100				DSM1884		This study
*E. coli* Top 10/pAJ100 – Region D				DSM1907		This study
AB5075 *att*Tn*7*-*hph*	AB5075 with a hygromycin resistance gene in the *att*Tn*7* site			DSM1926		This study
AB5075 *copR*/*cusR*106:T26 *att*Tn*7*-*hph*	The *cusR* mutant strain with a hygromycin resistance gene in the *att*Tn*7* site			DSM1927		This study
AB5075 *copC*160:T26 *att*Tn*7*-*hph*	The *copC* mutant strain with a hygromycin resistance gene in the *att*Tn*7* site			DSM1928		This study

*^1^For simplicity, mutant strains that were found to have increased copper sensitivity and were included in multiple assays were given a shorthand name. ^2^The mutant strains in the transposon library were created using a Tn*5* transposon carrying a tetracycline resistance cassette (T26) or hygromycin resistance cassette (T101) (Gallagher et al., 2015). Individual strains were purchased from the ordered, annotated library. The transposon’s position in the ORF is indicated as a base pair position out of the total base pairs (denoted in parenthesis) in the ORF; this information was taken directly from the mutant library list (Gallagher et al., 2015). ^3^Complemented strains were created by tri-parental mating of each transposon mutant strain with *E. coli* DH5α carrying the appropriate ORF and native promoter on the plasmid pUC18T mini-Tn*7*-*hph*, as well as with the two helper strains of *E. coli* that aid in the mating and transposition processes. The Tn*7* from the pUC18T mini-Tn*7*-*hph* plasmid, containing a hygromycin resistance gene and the desired complementation ORF and promoter, inserts into the AB5075 chromosome at the *att*Tn*7* site located in an intergenic region downstream of the *glmS* locus. MCS = multiple cloning site.*

### AB5075 Mutant Strains

Transposon mutant strains were purchased from the comprehensive, ordered transposon library engineered by Dr. Colin Manoil’s group at the University of Washington (Gallagher et al., 2015). In general, we chose strains with transposon insertions near the 5′ end of the gene; most have insertions in the first third of the ORF (Table 1). The isolates were received as stabs, which were streaked onto LB agar plates. The following day, a swab of the growth was used to inoculate an overnight culture in LB medium, which was subsequently frozen as a master stock for each mutant strain. To verify the desired mutations, each master stock was streaked on LB agar containing 10 μg/mL tetracycline to select for cells containing the Tn*5* insertion that characterizes the transposon library strains. For each, a single opaque colony was picked and restreaked onto LB agar containing tetracycline. The following day, a single opaque colony was picked and used to inoculate an overnight culture in LB medium containing 10 μg/mL tetracycline; the resulting cultures were frozen as working stocks for each mutant strain and were used for all future experiments. The desired transposon insertion was confirmed by PCR using primers that flank the proper ORF; primers are listed in Table 2. Throughout the manuscript, strains are always organized by ascending gene number, as in Figure 1. A few genes’ annotated names differ from the conventional names for the given protein; these genes’ annotated name and conventional name are both provided here, but the conventional names are used throughout for consistency and clarity: *actP2*/*copA1*, *copA*/*cueO*, *copR*/*cusR*, *copS*/*cusS*, *actP1*/*copA2*.

**TABLE 2:** Oligonucleotides used in this study.

**T26 insertion confirmation primers**			
**Flanked gene**	**Annotation**^1^	**Primer name**^2^	**Sequence (5′–3′)**
ABUW_0265		ABUW_0265-up ABUW_0265-dw	GTGCAATTTCTAACAGCCATG CAATAAATAAAAGATGCGGTGGG
ABUW_0266	*czcC*	czcC-up czcC-dw	TGGCAGCAATTACTCCAACC GCCATCCCGACTTATTGAGCT
ABUW_0267	*czcB*	czcB-up czcB-dw	CACCAATCCACGCCAGAAC TGGCGAAAGATGCCTTAATGC
ABUW_0268	*czcA*	czcDA-up czcA-dw	GCAACAGTAGTCGCATTTGTGG GCTTCTTACTGAAGTCCGAATTGG
ABUW_0269	*czcD*	czcDA-up czcD-dw	GCAACAGTAGTCGCATTTGTGG GGCAACAGTGGTTATTGGTGG
ABUW_0611		ABUW_0611-up ABUW_0611-dw	GGTTTCTAGGAGCGCTGCC GCACCTGCAAAGTATGGCTCTAC
ABUW_2705		ABUW_2705-up ABUW_2705-dw	CATGACAGCAGGTCTTAAACC CCTGTGGATCGAGGAGATATTAC
ABUW_2706	*cueR*	cueR-up-NEW cueR-dw-NEW	GAGGTGTGAGATGAATATCGGTCAG GTACTTGAGTGATGGCCGC
ABUW_2707	*copA1*	CopA2_up CopA2_dw	GCTTGACCTTCCCATGATGG CTGACCGATATTCATCTCACACCTC
ABUW_3226		ABUW_3226-up ABUW_3226-dw	GTATTATGGAGCTGGGTTAACAC GTTCCTCCAGTATGTGAAGAC
ABUW_3227	*pcoB*	CopB2-up CopB2-dw	CTGAAAATGAGAAAGGAGCTAG CATAAGCTTACTTTTTGTTCGGC
ABUW_3228	*pcoA*	CopA2-up CopA2-dw	CGTCCACCAAAAGCTTAATCC GAGAAAGCACCGCAACATATC
ABUW_3229		ABUW_3229-up ABUW_3229-dw	CCAAGATAAATAGATTGCTCAAGGC CGAGGCTGCTAATCGTTTTATG
ABUW_3230		ABUW_3230-up ABUW_3230-dw	GGATAACTGTGTTGATAACCATATGG CCATCTGAGTGGATTGTTGTTAAG
ABUW_3220	*copB*	CopB1-up CopB1-dw	CCTAATACTAATGATAAGGGGG CTTTATGAATAGTAGCTTCGGC
ABUW_3221	*cueO*	ABUW_3322-up CopA1-dw	CCGGTTCCAACCTATAAGTTAG GCAAAAGCTAGACCTGATAATCC
ABUW_3322		ABUW_3322-up ABUW_3322-dw	CCGGTTCCAACCTATAAGTTAG GTTAATTGAGGGCTAAGCAGG
ABUW_3323	*cusR*	CusR-up CusR-dw	CTTAATATGCGATACGGGGC GCAATACGGAAACTGATCGC
ABUW_3324	*cusS*	CusS-up CusS-dw	GGAATGGGGTATGTCTTAGAGG GAAAGGATCATTCTCTGACTACTC
ABUW_3325	*copA2*	CopA1_up_NEW CopA1_dw_NEW	GCCGGAGTAGCACTTTCAAG GAGGAAGGCACAGGTTTAGG
ABUW_3326	*copC*	CopC-up CopC-dw	GGAATTTCACTGGGTCATTAC CTGCTGGCATATAGACTAC
ABUW_3327	*copD*	CopD-up CopD-dw	GCATATTACTATCGCCGG GAAAGCATTGCTACTACTCC

**Complementation cloning primers**			

**Fragment**	**Primer name**	**Sequence (5′–3′)**^3^	

*pcoAB*promoter	pcoAB promoter up	CCCGGTACCcggtagtgtagacgcttaa	
	pcoAB promoter dw-A	GGCTGCTAATCGTTTTATGAGACATtaatgatctaggtctcttactaaaa	
	pcoAB promoter dw-B	AGAAAATAACTTAGTGATGCGCATtaatgatctaggtctcttactaaaa	
*pcoA* ORF	pcoA complement up	TTTTAGTAAGAGACCTAGATCATTAatgtctcataaaacgattagcagcc	
	pcoA complement dw	CCCGGATCCgagaaagcaccgcaacatatc	
*pcoB* ORF	pcoB complement up	TTTTAGTAAGAGACCTAGATCATTAatgcgcatcactaagttattttct	
	pcoB complement dw	CCCGGATCCcatctttgcaccataactgacac	
*copAB* promoter	copAB promoter up	CCCGGTACCaagagcttgatgtttacctg	
	copAB promoter dw	cattgattgctccaaaaataaattttatataactaac	
*cueO* ORF	NEW copA/cueO complement up	AAATTTATTTTTGGAGCAATCAatgtctagaaaattaagtcatgtcc	
	copA/cueO complement dw	CCCGGATCCccatgctttgaacatccgtatc	
*copB* ORF	NEW copB complement up	AAATTTATTTTTGGAGCAATCAatgcgcaccactaaaaaaatatattc	
	copB complement dw	CCCGGATCCgatttggaacgcttttaagccc	
*cusRS* promoter and *cusR* ORF	cusR complement up	CCCGGTACCcccaacacacataaaagcag	
	cusR complement dw	CCCGGATCCcttctagctgagtacggtc	
*cusRS* promoter	cusR complement up	CCCGGTACCcccaacacacataaaagcag	
	NEW cusRS promoter dw – cusS SOE	GATCGCATTAAACAGTTTATGAGTcatttattaagccccgtatcgcatattaag	
*cusS* ORF	cusS complement up	AATATGCGATACGGGGCTTAATAAatgactcataaactgtttaatgcgatc	
	cusS complement dw	CCCGGATCCgaaaggatcattctctgactactc	
*copCD* promoter and *copC* ORF	copCD promoter up	CCCGGTACCccatgccacagacaggatc	
	copC complement dw	CCCGGATCCcgtcccagtagaaaatagctaaagg	
*copCD* promoter	copCD promoter up	CCCGGTACCccatgccacagacaggatc	
	copCD promoter dw	catttcatcacctttttaaaattagtacttgc	
*copD* ORF	copD complement up	GTACTAATTTTAAAAAGGTGATGAAatgaatcctgaaacttggatatatgcaac	
	NEW copD comp dw	CCCGGATCCgacgtggtccttttatgcagc	
**Primers used in the creation of pAJ100**			
pWHori *Nco*I For	GATTACACCATGGgatcgtagaaatatctatgattatcttgaa		
pWHori *Nco*I Rev	GATTACACCATGGggattttaacattttgcgttgttccaa		

*^1^Gene names are given where available. For those whose annotated name is different from the name traditionally given to that protein, the conventional name is listed. ^2^The primer name may not match the gene name. ^3^Capital letters signify added primer tail sequences for SOE or restriction site. Underlined sequences are restriction enzyme cut sites.*

### Generation of Complemented Strains

The previously described Tn*7*-based strategy of complementation was used to complement the transposon mutant strains (Kumar et al., 2010; Jacobs et al., 2014a, b). The wild-type ORF and native promoter were amplified using the primers listed in Table 2. If the ORF was not located directly downstream of the promoter due to operonic structure, the two DNA fragments were fused by SOE PCR. The full construct was amplified with Phusion Hot Start Polymerase (Thermo Fisher Scientific) and then cloned into pUC18T mini-Tn*7*-*hph* using the added restriction enzyme sites (Table 2). The resulting plasmids were transformed into electrocompetent DH5α *E. coli* and transformants were selected for on LB agar with 100 μg/mL ampicillin. Correct plasmid construction was confirmed by digestion and then the resulting *E. coli* strains were mated with the AB5075 transposon mutant strains using the tri-parental protocol described by Jacobs et al. (2014b). Briefly, 100 μL of four overnight cultures (*E. coli* carrying the complementation construct in pUC18T-mini Tn*7*-*hph*, two helper strains, and the AB5075 mutant strain) grown in LB medium with the appropriate antibiotics were mixed, washed twice, and spotted onto plain LB agar. The mating was allowed to proceed for 1 to 24 h. The cells in the spot were collected with a sterile loop and plated on LB agar with 25 μg/mL chloramphenicol to kill the *E. coli* strains and 250 μg/mL hygromycin to select for *A. baumannii* with the Tn*7* insertion. Resulting colonies were screened for Tn*7* insertion using colony PCR with the previously described *att*Tn*7* primers (Jacobs et al., 2014a). Single colonies were restreaked onto LB agar with 10 μg/mL tetracycline and 250 μg/mL hygromycin. A single opaque colony was used to inoculate an overnight culture in LB with tetracycline and hygromycin, which was ultimately used to create a freezer stock of the strain. The correct insertion at the *att*Tn*7* site was confirmed by PCR and sequencing. All of the strains carried the Tn7 insertion in the same direction: forward, relative to *glmS*.

### Cloning of Region D From AB5075 Into Other *A. baumannii* Strain Backgrounds

#### Creation of pAJ100

An *E. coli–A. baumannii* shuttle vector with a hygromycin resistance gene was created to move genes into multidrug-resistant *A. baumannii*. To generate this vector, pMQ300 (Kalivoda et al., 2011) was digested with *Bsp*HI to remove the 1.7 kb region that contained yeast replication genes URA3 and CEN6/ARSH4. The *A. baumannii* origin of replication of pWH1266 (Hunger et al., 1990) was amplified using the pWHori *Nco*I For and pWHori *Nco*I Rev primers described in Table 2. This insert was digested with *Nco*I and then ligated to the remaining 4.7 kb of pMQ300. The resulting 6 kb plasmid was transformed into *E. coli* and plated on LB agar with 250 μg/mL hygromycin. The resulting vector was named pAJ100. This vector has a *lacZ* insertion site for genes that contains cut sites for the following enzymes: *Sma*I, *Bam*HI, *Hin*dIII, *Kpn*I, *Pst*I, and *Pvu*I.

#### Creation of pAJ100 – Region D

Region D was amplified from AB5075 chromosomal DNA with Phusion Hot Start Polymerase (Thermo Fisher Scientific) using the copB complement dw and copD complement dw primers described in Table 2. The fragment was cloned into pAJ100 using the added *Bam*HI restriction enzyme sites. The resulting pAJ100-Region D plasmid was electroporated into *E. coli* Top 10 cells, which were plated on 250 μg/mL hygromycin, 40 μg/mL X-gal, and 1 mM IPTG. A white colony was restreaked for isolation, and a single colony was used to inoculate an overnight culture of LB medium with 250 μg/mL hygromycin. Plasmid was purified and then electroporated into AB4857 and AB5711, which had been made electrocompetent using the protocol described by Jacobs et al. (2014b). Transformants were selected for on LB with 250 μg/mL hygromycin. Double purified single, opaque colonies were used to inoculate overnight cultures of LB medium with 250 μg/mL hygromycin, which were then used to create freezer stocks of the strains. Correct plasmid construction was confirmed by digestion and PCR.

### Determination of *A. baumannii* Sensitivity to Copper in Liquid Culture

**FIGURE 2:**
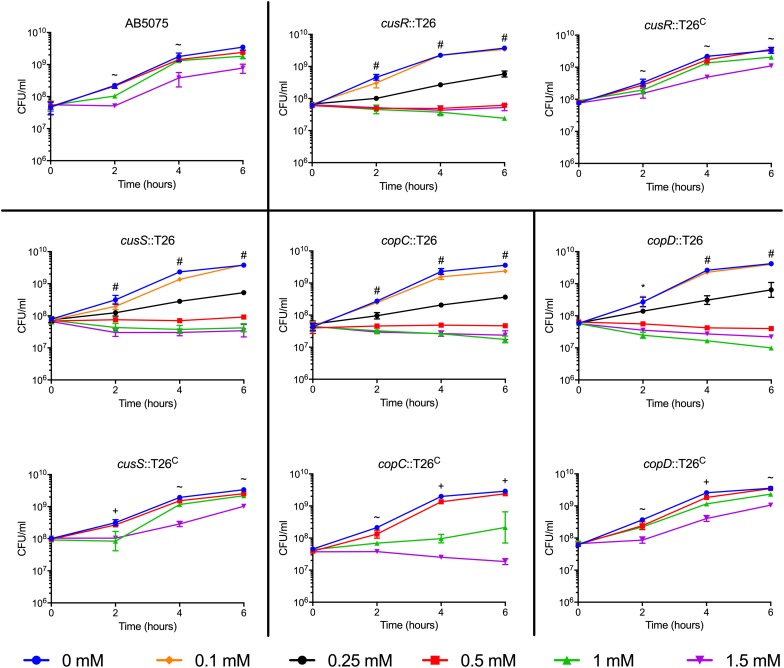
Growth of highly copper sensitive AB5075 mutant and complementation strains. Strains were grown for 6 h at 37°C in M9 medium supplemented with the concentration of CuSO_4_ indicated in the legend. Growth was measured both by turbidity (OD_600_, data not shown) and enumeration of viable colonies (CFU/mL). Black lines are used to group the mutant strain with the corresponding complemented derivative. The data are presented as geometric mean and SEM of three biologically independent experiments. Two-way ANOVA with Tukey’s adjustment for multiple comparisons was used to compare growth in each copper concentration at each timepoint. The symbols indicate which copper treatments were statistically different (*P* < 0.05) from the 0 mM control, as follows: ∼, growth in 1.5 mM CuSO_4_ was different from the control; +, growth in 1 and 1.5 mM CuSO_4_ was different from the control; *, growth in 0.5, 1, and 1.5 mM CuSO_4_ was different from the control; and #, growth in 0.25, 0.5, 1, and 1.5 mM CuSO_4_ was different from the control.

**FIGURE 3:**
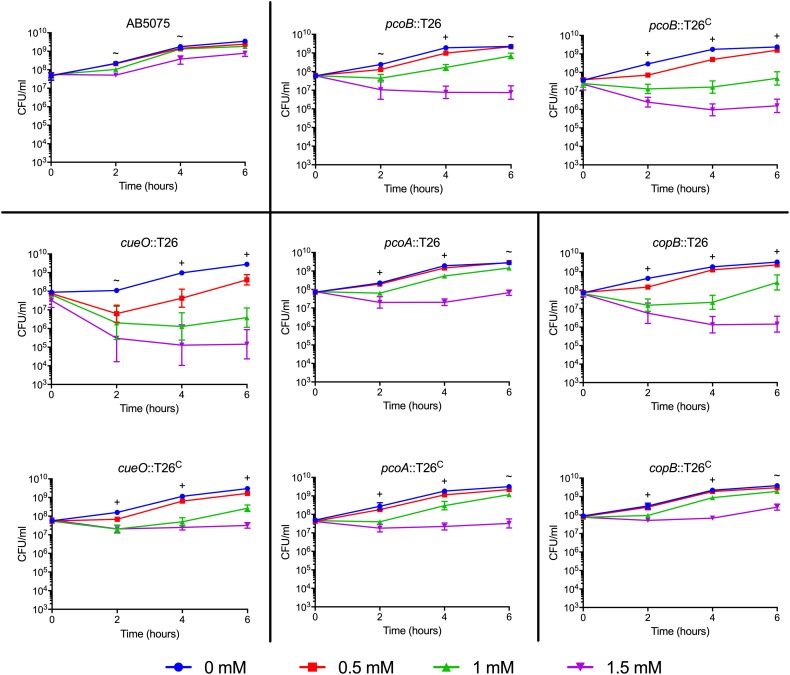
Growth of moderately copper sensitive AB5075 mutant and complementation strains. Strains were grown for 6 h at 37°C in M9 medium supplemented with the concentration of CuSO_4_ indicated in the legend. Growth was measured both by turbidity (OD_600_, data not shown) and enumeration of viable colonies (CFU/mL). Black lines are used to group the mutant strain with the corresponding complemented derivative. The data are presented as geometric mean and SEM of three biologically independent experiments. Two-way ANOVA with Tukey’s adjustment for multiple comparisons was used to compare growth in each copper concentration at each timepoint. The symbols indicate which copper treatments were statistically different (*P* < 0.05) from the 0 mM control, as follows: ∼, growth in 1.5 mM CuSO_4_ was different from the control; +, growth in 1 and 1.5 mM CuSO_4_ was different from the control.

To assess the effect of copper on *A. baumannii* growth, bacterial strains were grown in the presence of increasing concentrations of copper sulfate as previously described (Williams et al., 2016; Williams and Merrell, 2019). Briefly, bacteria from overnight cultures were subcultured at an optical density (OD_600_) of 0.05 in 10 mL of M9 medium containing 0.1–1.5 mM CuSO_4_ (Aldrich, St. Louis, MO, United States). Growth was measured every hour for 6 h by optical density (OD_600_) and by enumerating CFU every other hour. Three biologically independent experiments were completed for each strain showing noticeable copper sensitivity (Figures 2, 3); if no difference from the wild-type was observed, two biologically independent experiments were completed (Supplementary Figure S1). To ensure that the excess sulfate found in the copper sulfate was not responsible for the growth phenotypes, sodium sulfate was also tested at 1.5 mM using the same growth conditions; CFU were enumerated at 0 and 4 h. Three biologically independent experiments were completed (Supplementary Figure S2).

### Assessing Copper Sensitivity of *A. baumannii* Biofilm

Biofilms were grown, exposed to copper, and assessed for survival as previously described with minor adjustments to the incubation time and growth media (Williams et al., 2016). Briefly, overnight cultures of *A. baumannii* strains were grown in CFA medium (Evans et al., 1977), and bacteria were diluted to an OD_600_ of 0.05 in 24-well tissue-culture treated plates (Corning, Corning, NY, United States) in 1 mL of CFA medium. Plates were incubated statically at 37°C for 24 h. To expose the pre-formed biofilms to copper, the broth was removed and was replaced with 1.5 mL of M9 medium containing 1.5 mM CuSO_4_; biofilms were incubated for another 6 or 24 h. To determine the number of planktonic cells, at each timepoint (0, 6, and 24 h) the medium was transferred to a 1.5 mL Eppendorf tube and vortexed for approximately 5 s to disrupt any clumped bacteria in the supernatant; samples were visually inspected to confirm the absence of clumped bacteria. To determine the number of biofilm cells, the biofilm was scraped from the sides and bottom of the well with a pipette tip and was resuspended in 1 mL of PBS. Samples of both planktonic cells and biofilm cells were plated to enumerate CFU. Three biologically independent experiments were performed.

### Quantification of Intracellular Copper via ICP-MS

#### Sample Collection

To measure the intracellular copper concentration, dry cell samples were collected and analyzed by inductively coupled plasma mass spectrometry (ICP-MS). Overnight cultures were subcultured to an OD_600_ of 0.05 in a large 100 mL volume. The subcultures were grown for 2.5 h to reach log phase, and a time zero (T0) sample was collected. The sample collection procedure was as follows: 6 mL of culture was pelleted in three 2-mL tubes using a 4°C table top centrifuge at max speed for 30 s; the supernatant was aspirated. The three pellets were resuspended in 1 mL of cold wash solution (PBS with 0.5 mM EDTA) in a single tube and pelleted again; the supernatant was aspirated. The wash steps were repeated for a total of three washes. After the final aspiration, the tube was left open to begin air drying. A 0.5 mL sample of the culture was separately collected in a cuvette to measure the OD_600_ of the culture. The cell pellets were fully dried in an 80°C SpeedVac vacuum concentrator for 1 h and stored at −20°C. After collection of the T0 sample, 0.25 mM copper sulfate was added directly to the large culture, swirled immediately, and returned to the shaking incubator. Additional samples were collected along the time course. In experiment two, with the complete set of mutant strains showing a copper sensitive phenotype, the final wash step was conducted in a 15-mL conical tube and spun for 3 min at 4150 rpm/3,700 × *g*, and the cell pellets were dried overnight in a 37°C incubator. This eliminated the need for transfer of many samples to conical tubes before ICP-MS analysis.

After the copper shock portion of the experiment was completed, the remaining culture was washed and placed into fresh growth medium for recovery. Briefly, the remaining culture was placed in a 50-mL conical tube and spun for 15 min at maximum speed (4150 rpm/3,700 × *g*) to pellet the cells. The cells were resuspended in 2 mL of wash solution and were transferred to two 2-mL tubes. The cells were pelleted by spinning for 1 min at max speed in a tabletop centrifuge; the supernatants were aspirated. The wash steps were repeated for a total of three washes. The cells were resuspended in 50 mL of pre-warmed growth medium without added copper. Samples were collected in the same way as described above across the recovery time course. Three biologically independent replicates were performed for both experiments.

#### Copper Measurement

The dry cell pellets were thawed at room temperature and dissolved in 200 μL of concentrated HNO_3_ (trace metal grade) overnight at 80°C. The following day, the samples were cooled to room temperature and diluted with 2 mL of MilliQ water, for a final HNO_3_ concentration of 6%. Where necessary, the samples were transferred to metal-free 15-mL conical tubes for ICP-MS analysis. The metal content of the samples was then analyzed by quantitative ICP-MS. Copper concentrations were determined by injecting diluted samples into an Agilent 7700x ICP-MS (Agilent Technologies, Santa Clara, CA, United States). Copper levels were detected using an Octopole Reaction System cell (ORS) in He mode. The ICP-MS parameters used for the analysis were: an RF power of 1550 W, an argon carrier gas flow of 0.99 L/min, helium gas flow of 4.3 mL/min, octopole RF of 200 V, and OctP bias of −18 V. Samples were directly infused using the 7700x peristaltic pump with a speed of 0.1 rps and a micromist nebulizer. Copper concentrations in samples were derived from a calibration curve generated by a series of dilutions of a copper atomic absorption standard (Fluka Analytical, St. Louis, MO, United States) prepared in the same matrix as the samples. Data analysis was performed using Agilent’s Mass Hunter software (4.4 version).

#### Calculations

The value originally obtained from ICP-MS (ppb, μg/L) was divided by the molecular weight of copper (63.546 g/mol) to determine the concentration of copper in the diluted sample (μM). The sample concentration was multiplied by the total volume (2.2 mL) to determine the quantity of copper in the sample (nmol). The total amount of copper was divided by the sample volume (6 mL) to determine nmol/mL of cells and divided by the OD_600_ to determine the nmol/ODU of cells. The data was plotted as nmol/ODU vs. time. This protocol was recently described in more detail as a methods chapter (Williams et al., 2019).

### Infection of *Galleria mellonella* Caterpillars

*Acinetobacter baumannii* strains were grown overnight in LB medium with the appropriate antibiotics: tetracycline for transposon mutant strains, and both tetracycline and hygromycin for complemented strains. Overnight cultures were diluted to an OD_600_ of 0.05 and were grown 3 h to log phase. The cells were adjusted in PBS to a specific OD_600_ to achieve the desired CFU/mL, and were then diluted 1:10 in PBS with 0.01% bromophenol blue dye for visibility. For a dose of 5 × 10^4^ CFU, bacteria were adjusted to an OD_600_ of 0.2–0.3, which corresponded to 10^7^ CFU/mL; for a dose of 5 × 10^5^, bacteria were adjusted to an OD_600_ of 2–3, or 10^8^ CFU/mL. The exact number of bacterial cells in the inoculum was determined by serial dilution and plating for enumeration of CFU. The actual doses ranged from 3.53–9.63 × 10^4^ to 3.38–9.60 × 10^5^.

*Galleria mellonella* larvae (Vanderhorst Wholesale, Inc., Saint Marys, OH, United States) were utilized within 4 days of receipt. Larvae weighing 200–300 mg were used. The injections were carried out as described previously (Jacobs et al., 2014a) with minor changes. Briefly, 5 μl of the sample was injected into the last left proleg using a 10-μl glass syringe (Hamilton, Reno, NV, United States) fitted with a 31G needle. Each experiment included control groups of non-injected larvae and PBS-injected larvae. All larvae were incubated at 37°C, and death was assessed at 24 h intervals for 5 days. Larvae were considered dead if they didn’t respond to physical stimulus. All larvae that progressed to the pupation stage of the life cycle were excluded. For consistency, wild-type, mutant strain, and complemented strain infected groups that are plotted together were injected in the same experiment. Experiments were repeated using two or three different orders of larvae with 12 to 18 larvae per experimental group; the total n ranged from 25 to 48 larvae per strain. A single data set was excluded in which the wild-type strain killed only 30% of the caterpillars; no data from this set of infections was used.

### Murine Pneumonia Infection Model

Mice were infected with *A. baumannii* as described previously (Jacobs et al., 2014a). Briefly, 6-week-old female BALB/c mice were first rendered temporarily neutropenic by intraperitoneal injection with cyclophosphamide on days −4 and −1 before infection. On day 0, mice were anesthetized with 2–5% isoflurane gas and inoculated intranasally with approximately 5 × 10^6^ CFU of *A. baumannii* in a total volume of 25–50 μL PBS. Animals were monitored for morbidity and mortality for 7 days, and humanely euthanized with CO_2_ inhalation when necessary. All mouse studies were conducted in accordance with the *Guide for the Care and Use of Laboratory Animals* (National Research Council, 2011), and procedures were approved by the Institutional Animal Care and Use Committee at the Walter Reed Army Institute of Research (protocol 16-BRD-48S). Experiments with the *cusR* mutant strain and complemented derivative were performed in four biological replicates with five mice per group (*n* = 20 per strain), spaced two and two over time to ensure reproducibility; two biological replicates were completed with the *copD* mutant strain and complemented derivative (*n* = 10 mice per strain).

### Data Analysis and Statistics

All graphs and statistical analyses were carried out using GraphPad Prism 7 (GraphPad Software, Inc., La Jolla, CA, United States). A two-way analysis of variance (ANOVA) with Tukey’s correction for multiple comparisons was used to evaluate differences in growth in various copper treatments. Data are presented as the geometric mean and standard error of the mean. A two-way ANOVA with Sidak’s or Dunnett’s correction was used to compare intracellular copper concentrations. Data are presented as arithmetic mean and range. An ordinary one-way ANOVA with Dunnett’s correction followed by linear contrasts with a Bonferroni adjustment was used to compare biofilm and planktonic cells percent survival at each concentration and time point to the wild-type. Data are presented as arithmetic mean and range. Survival curves were compared using the Mantel–Cox log rank test with Holm’s correction for multiple comparisons. For all tests, a two-sided α level set at 0.05 determined significance.

## Results

### Mutant Strains of AB5075 Exhibit Copper Sensitivity

Many putative copper-related genes have been identified in *A. baumannii*; however, their predicted functions are based solely on protein homology and the role of these proteins in copper resistance is largely unknown. To investigate the contribution of individual genes to copper resistance in AB5075, we used the available arrayed transposon mutant strain library (Gallagher et al., 2015) to initiate our studies. To this end, we obtained 21 mutant strains, each of which contained a T26 transposon insertion in a putative copper resistance gene. Specifically we targeted the 22 genes found in regions A–D that we previously identified as putative copper resistance genes in AB5075 (Williams et al., 2016); only one small ORF, ABUW_2708, was not represented in the available mutant strain library. To assess the role of the remaining 21 genes in copper resistance, each of the mutant strains was individually grown in M9 medium containing increasing concentrations of CuSO_4_. Thirteen of the mutant strains did not differ from the wild-type in their ability to grow in the presence of copper, indicating that the mutated gene was not individually contributing to copper resistance in the tested conditions (Supplementary Figure S1). Conversely, eight of the mutant strains showed significant changes in copper sensitivity relative to the wild-type strain. The phenotypes of these eight strains fell into two categories: moderately and highly sensitive. Strains bearing mutations in *cusR*, *cusS*, *copC*, and *copD* were all highly sensitive to copper and showed significant growth defects in as little as 0.25 mM CuSO_4_ (Figure 2). In contrast, strains bearing mutations in *pcoB, pcoA, copB*, and *cueO* were moderately copper sensitive and only displayed increased sensitivity relative to the wild-type strain in the presence of at least 1 mM CuSO_4_ (Figure 3). Of note, nearly all the mutant strains with copper sensitive phenotypes contained mutations in genes carried in region D (Figure 1), and these strains displayed the most substantial defects in copper resistance.

To ensure that excess sulfate found in the copper sulfate was not responsible for the growth phenotypes, we performed a similar growth experiment with 1.5 mM sodium sulfate; sodium sulfate did not inhibit growth of the wildtype or mutant strains (Supplementary Figure S2). To confirm that the copper sensitive phenotype was due to the T26 transposon insertion, we constructed a complemented derivative of each of the eight mutant strains of interest. Complementation was achieved by insertion of a wild-type copy of the ORF and its native promoter in an *att*Tn*7* site downstream of the *glmS* locus (Kumar et al., 2010). Each of the complemented strains was individually tested in the same growth experiment. Complete restoration of copper resistance to wild-type levels was observed in the *cusR*, *cusS*, and *copD* complemented strains (Figure 2). Partial restoration of copper resistance was observed in the *copB*, *cueO*, and *copC* complemented strains (Figures 2, 3). No functional complementation was observed in the *pcoA* and *pcoB* complemented strains (Figure 3); the complemented derivative of the *pcoB* mutant strain actually grew slightly less than its parent strain in 1 mM copper sulfate. The lack of complementation perhaps suggests that the identified phenotypic changes are not purely due to the transposon insertion in *pcoA* and *pcoB*, or that this complementation strategy was insufficient for these two particular genes. Taken together, our mutational analyses indicate that many of the identified putative copper resistance genes indeed contribute to copper resistance in *A. baumannii*. Moreover, the results suggest that the genes found in region D are crucial for the highest levels of copper resistance.

### Region D Enhances Copper Resistance in Other *A. baumannii* Strains

**FIGURE 4:**
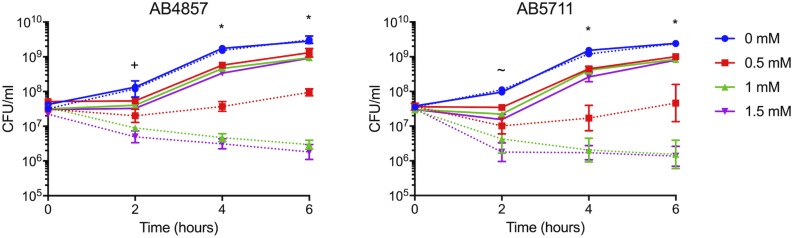
Growth of AB4857 and AB5711 carrying Region D. Region D from AB5075 was cloned into pAJ100 and transformed into AB4857 and AB5711, which do not naturally encode the region D genes. Strains carrying the empty vector, pAJ100, were used as controls. The strain background is indicated in the graph title (AB4857, left; AB5711, right); strains carrying the control vector pAJ100 are indicated with dotted lines, and strains carrying pAJ100-Region D are indicated with solid lines. Strains were grown for 6 h at 37°C in M9 medium supplemented with the indicated concentration of CuSO_4_. Growth was measured both by turbidity (OD_600_, data not shown) and enumeration of viable colonies (CFU/mL). The data are presented as geometric mean and SEM of three biologically independent experiments. Two-way ANOVA with Tukey’s adjustment for multiple comparisons was used to compare growth of the strain carrying the pAJ100 vector to the strain carrying pAJ100-Region D in each copper concentration at each timepoint. The symbols indicate how many comparisons were statistically significant (*P* < 0.05) at each timepoint, as follows: ∼, growth in 1.5 mM CuSO_4_ was different between the two strains; +, growth in 1 and 1.5 mM CuSO_4_ was different between the two strains *, growth in 0.5, 1, and 1.5 mM CuSO_4_ was different between the two strains.

Our previous analysis of multiple clinical isolates of *A. baumannii* identified two isolates, AB4857 and AB5711, that both showed inherently reduced copper resistance as compared to AB5075. Notably, neither of these strains carried the genes from region D in their genomes (Williams et al., 2016). Given our finding that mutations in the genes located in region D caused the greatest copper sensitivity in AB5075 (Figure 2), we hypothesized that the transfer of region D to AB5711 and AB4857 would increase their overall level of copper resistance. Indeed, when region D from AB5075 was carried on the plasmid pAJ100 in these strains, pAJ100-Region D conferred increased copper resistance comparable to the level seen in AB5075; the empty pAJ100 vector did not affect resistance (Figure 4). Thus, these results also indicate that the genes in region D are important for high levels of copper resistance in *A. baumannii*.

### Biofilms of Mutant Strains Retain Increased Copper Sensitivity

**FIGURE 5:**
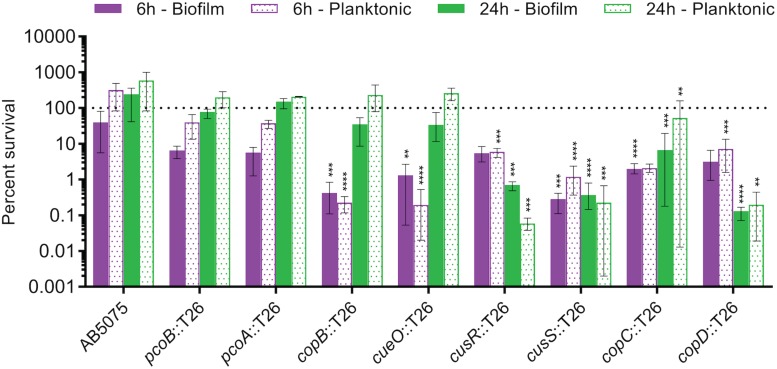
Copper-dependent killing of AB5075 mutant strain biofilms and planktonic cells. Cultures were statically incubated in a 24-well tissue culture-treated plate at 37°C to establish biofilms. After 24 h, the broth was replaced with M9 medium containing 1.5 mM CuSO_4_. After an additional 0, 6, or 24 h of incubation, bacteria from both the supernatant (planktonic) and the biofilm were plated for enumeration of CFU. Percent survival was calculated as the percentage of bacteria present at time zero under each condition. Data are presented as mean and range for three biologically independent experiments. One-way ANOVA with Dunnett’s correction was used to compare each mutant strain to the wild-type at each indicated condition. Statistically significant comparisons are indicated with the asterisks, as follows: **P < 0.01; ***P < 0.001; ****P < 0.0001.

Biofilm formation is an important component of the *A. baumannii* lifecycle; the bacterium is known to create biofilms on both biotic and abiotic surfaces, which aides in fomite-based transmission and in virulence (Longo et al., 2014; Zurawski et al., 2019). Moreover, biofilms are notorious for their ability to decrease the effectiveness of antibiotic treatment (Hoiby et al., 2010). Because any future copper-based therapeutics would likely need to be effective against *A. baumannii* found within a biofilm structure, we next tested the copper sensitivity of biofilms formed by the copper sensitive mutant strains as compared to wild-type AB5075. Biofilms were established in complex media for 24 h before switching the medium to M9 medium supplemented with 1.5 mM CuSO_4_. CFU were enumerated from both the biofilm and supernatant at 0, 6, and 24 h post copper shock, and data were expressed as percent survival relative to T0. For the wild-type AB5075, a slight decrease in biofilm CFU was observed at 6 h, but no obvious sensitivity to copper was observed in the planktonic population at this timepoint or in the biofilm or planktonic populations at 24 h (Figure 5). While the *pcoA* and *pcoB* mutant strains of region B did not demonstrate survival differing from the wild-type strain in these biofilm conditions, the strains bearing mutations in region D (*copB*, *cueO*, *cusR*, *cusS*, *copC*, *copD*) each displayed statistically significant decreases in survival of both biofilm and planktonic cells relative to the wild-type strain; a 1–3 log loss in recoverable CFU was observed. The *copB* and *cueO* mutant strains displayed an early defect but recovered to wild-type levels by 24 h. Conversely, the *cusR*, *cusS*, *copC*, and *copD* mutant strains did not recover. These data demonstrate that most mutant strains retain copper sensitivity relative to the wild-type strain even within a biofilm structure, suggesting that future copper resistance targeting therapeutics would likely not be hindered by any inherent copper resistance of a biofilm.

### Copper-Sensitive Mutant Strains Accumulate More Intracellular Copper

**FIGURE 6:**
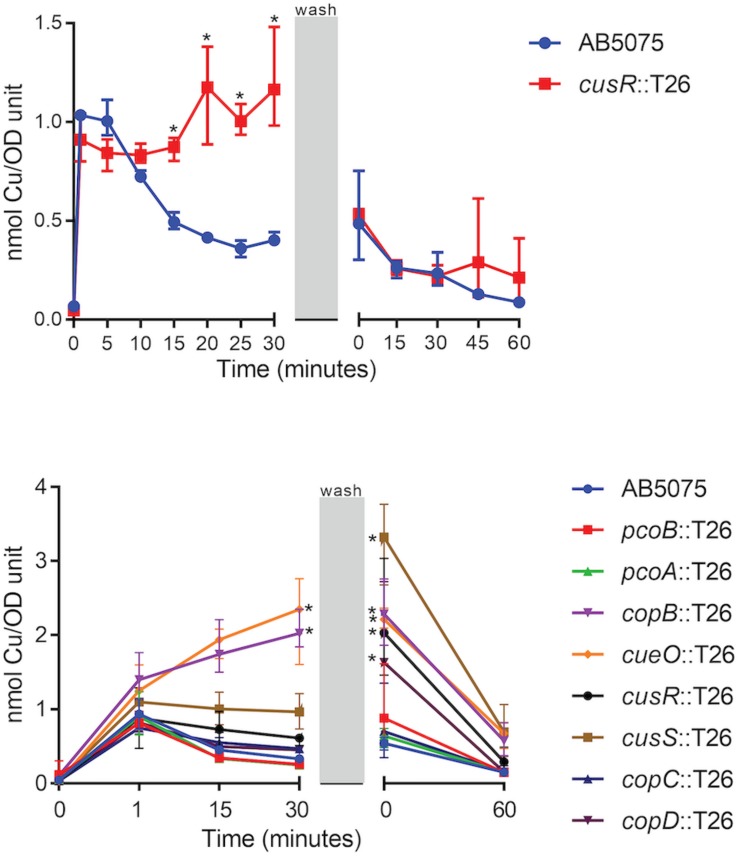
Accumulation of intracellular copper. Log phase cultures were treated with 0.25 mM CuSO_4_ and samples were collected at the indicated timepoints. During the wash phase, the remaining culture volume was washed and resuspended in fresh M9 medium without supplemented copper. Cell samples that were collected at each timepoint were washed and dried, and copper content was measured by ICP-MS. Data are presented as the mean and range for three biologically independent experiments. Two-way ANOVA with Sidak’s correction was used to compare the *cusR* mutant strain to the wild-type at all timepoints (top); two-way ANOVA with Dunnett’s correction was used to compare all the mutant strains to wild-type at all timepoints (bottom). Statistically significant comparisons are indicated with an asterisk (*P* < 0.01). At 0 min post-wash, five strains were significantly different from wild-type: *copB*, *cueO*, *cusR*, *cusS*, and *copD* (bottom).

To begin to understand the ability of the wild-type strain to adapt to copper stress as well as the nature of the copper homeostasis defects in the copper sensitive mutant strains, we utilized ICP-MS to temporally measure copper accumulation in strains exposed to a copper shock (0.25 mM). Previous studies in other bacteria have demonstrated that intracellular copper accumulates in mutant strains bearing mutations in copper-related proteins, including *pcoB*, *pcoA*, *copA*, *cusR*, and *cusS* (Outten et al., 2001; Lee et al., 2002; Gonzalez-Guerrero et al., 2010; Gudipaty et al., 2012). To optimize the assay, we focused our initial efforts on wild-type AB5075 and the *cusR* mutant strain. In wild-type AB5075, the intracellular copper concentration increased ∼10x one minute following copper exposure. However, the level reduced to ∼5x in approximately 15 min and fully recovered to baseline levels 60 min following removal of excess copper from the media (Figure 6). Thus, in the wild-type strain, the cells appear to respond to copper stress via the deployment of efflux mechanisms that reduce the level of intracellular copper. In comparison, for the copper sensitive *cusR* mutant strain, intracellular copper levels immediately increased ∼10x, but showed no reduction while copper was present. However, after copper removal, the level similarly recovered to baseline within 60 min (Figure 6). These data suggest that in the presence of excess copper, the *cusR* mutant cells are unable to control the intracellular copper concentration and cannot efflux copper ions as well as the wild-type cells.

Based on the above results, we modified the sampled timepoints and collection strategy in order to simultaneously test the wild-type and all eight of the mutant strains that previously demonstrated increased copper sensitivity (Figures 2, 3). Of note, the revised collection strategy resulted in a longer period of copper exposure as the washing steps took ∼20 min longer to collect the cells. Using this procedure, the *copB* and *cueO* mutant strains each showed significantly higher accumulation of copper relative to the wild-type strain during the 30 min copper shock. In addition, the *cusR*, *cusS*, and *copD* mutant strain accumulated a higher concentration of intracellular copper within the additional time of copper exposure found with the modified washing steps. All strains demonstrated recovery following the removal of the copper stress (Figure 6). Taken together, these results indicate that many of the mutant strains have defects in copper homeostasis and copper efflux, which likely contribute to their copper sensitivity.

### Copper Resistance Genes Are Important for Virulence in *Galleria mellonella* Larvae

**FIGURE 7:**
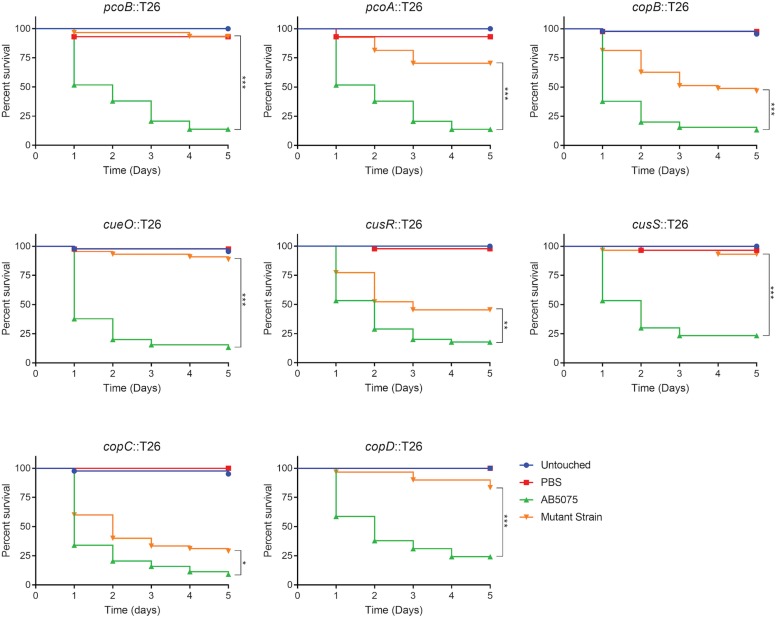
Survival of *Galleria mellonella* infected with AB5075 mutant strains. *G. mellonella* were injected with approximately 5.0 × 10^4^ CFU of the indicated strain and survival was monitored for 5 days. The gene interrupted in each mutant strain is indicated in the graph title. Experiments were repeated using two or three different orders of larvae with 12–15 larvae per experimental group; the total n ranged from 25 to 45 larvae per strain. Kaplan–Meier survival curves were compared (excluding Untouched and PBS controls) using the Mantel–Cox test with Holm’s correction for multiple comparisons. Statistically significant comparisons of the mutant strain to the wild-type strain are indicated by bars with asterisks signifying the *P*-value as follows: *P < 0.05; **P < 0.01; ***P < 0.001.

Given that copper is an important component of immune defense, we hypothesized that copper resistance is an important trait that affects virulence of *A. baumannii*. To begin to test this possibility, we assessed the ability of the copper sensitive mutant strains to kill *G. mellonella* caterpillars. This model was chosen because: (1) *G. mellonella* have been established as an inexpensive and simple infection model for many pathogenic bacteria, including *A. baumannii*, (2) these caterpillars can be maintained at 37°C, (3) these caterpillars have both humoral and cellular immune response pathways, and (4) this invertebrate model has been used for identification of bacterial virulence factors and virulence results often correlate with those obtained in mammalian models (Peleg et al., 2009; Jacobs et al., 2014a; Tsai et al., 2016). Groups of *G. mellonella* were individually infected with each strain of *A. baumannii*, incubated at 37°C, and observed for 5 days. Consistent with previous experiments with AB5075 (Jacobs et al., 2014a), at a dose of approximately 5.0 × 10^4^ CFU, wild-type AB5075 killed ∼85% of the *G. mellonella*, with most death occurring in the first 2 days post-infection (Figure 7). In contrast, the copper sensitive mutant strains were all attenuated in this model and killed significantly less *G. mellonella* than the wild-type strain (Figure 7). The *pcoB*, *pcoA*, *cueO*, *cusS*, and *copD* mutant strains were the most attenuated and killed less than 30% of the *G. mellonella*. The *copB* and *cusR* mutant strains were moderately attenuated and killed ∼55% of *G. mellonella*. The *copC* mutant strain was very mildly attenuated and killed ∼70% of *G. mellonella*. To ensure that the strains from the AB5075 transposon library did not show a general defect in the *G. mellonella* model, we additionally selected and tested five mutant strains that did not display a copper sensitive phenotype *in vitro*; none of these strains were attenuated for virulence in the *G. mellonella* model (Supplementary Figure S5).

As with the *in vitro* phenotypes, we also sought to functionally complement the virulence defects seen in the *G. mellonella* model. However, infection with the complemented strains resulted in similar rates of *G. mellonella* death as those infected with the corresponding mutant strain; in some cases, the complemented strains appeared slightly more attenuated (Supplementary Figures S3, S4). Based on these data, we speculated that the insertion at the *att*Tn*7* site was causing unintended attenuation in this model; to our knowledge, no published studies have shown functional complementation using the Tn*7*-based strategy in assays of *G. mellonella* survival. To test this possibility, a strain carrying an *att*Tn*7* insertion of only the hygromycin resistance gene was created and then compared in virulence to the wild-type background strain. Attenuation of the AB5075:Tn*7* strain relative to AB5075 was not consistently observed (Supplementary Figure S5). We reasoned that perhaps the effect was only evident in strains already carrying the T26 transposon insertion and that the size of the *att*Tn*7* insertion might be important. Thus, derivatives of the *cusR* and *copC* mutant strains were engineered to carry an *att*Tn*7* insertion of only the hygromycin resistance gene and were then compared to the parental mutant strain as well as the complemented derivatives, which each carried much larger *att*Tn*7* insertions. These hygromycin only insertion strains were both slightly more attenuated than the parental strains (Supplementary Figure S5). Furthermore, the complemented strains carrying the larger *att*Tn*7* insertion were even further attenuated (Supplementary Figure S5). These results suggest that the inability to functionally complement the mutant virulence phenotypes is due to the fact that insertion at the *att*Tn*7* site is attenuating in the *G. mellonella* survival model. The mechanistic reason for this effect is unknown, but may be due to disruption of an important genetic element near the *att*Tn*7* site, or perhaps polar effects from expression of the hygromycin resistance gene; all of our complemented strains contain insertions oriented in the same forward direction, therefore the hygromycin resistance gene and its promoter face out from Tn*7* and downstream of *glmS*. While the reason for this attenuation currently remains unclear, overall, the data strongly suggest that copper resistance, and many individual proteins in regions B and D, are specifically needed for full virulence of *A. baumannii* in the *G. mellonella* model. These conclusions are strengthened by a lack of attenuation of other transposon mutant strains that did not demonstrate increased copper sensitivity (Supplementary Figure S5), however, these conclusions would clearly be further strengthened by functional complementation in this model.

### CusR and CopD Are Important for Virulence in a Murine Pneumonia Infection Model

**FIGURE 8:**
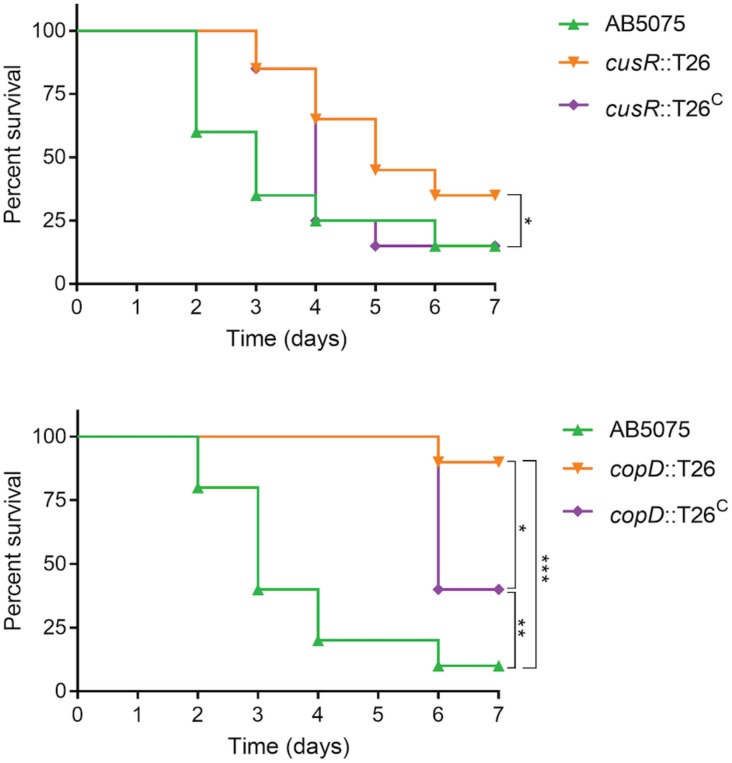
Assessment of virulence of AB5075 mutant and complemented strains in a murine pneumonia model. Mice were infected intranasally with approximately 5 × 10^6^ CFU of the indicated strain and survival was monitored for 5 days. Experiments with the *cusR* mutant strain and complemented derivative were performed in four biological replicates with five mice per group (*n* = 20 per strain), spaced two and two over time to ensure reproducibility; two biological replicates were completed with the *copD* mutant strain and complemented derivative (*n* = 10 mice per strain). Kaplan–Meier survival curves were compared using the Mantel–Cox test with Holm’s correction for multiple comparisons. Statistically significant comparisons are indicated by bars with asterisks signifying the *P*-value as follows: *P < 0.05; **P < 0.01; ***P < 0.001. The only significant comparison in the top panel is wild-type AB5075 vs. the *cusR* mutant strain.

Given the attenuated virulence observed in the *G*. *mellonella* model, we next wished to determine if *A. baumannii* copper resistance is also an important virulence determinant during infection of a mammalian host. To this end, we selected two of the mutant strains and their complemented derivatives to test in a murine pneumonia model; the *cusR* and *copD* mutant strains were selected due to their high level of copper sensitivity and successful *in vitro* complementation (Figure 2), as well as their phenotypic differences from the wild-type strain in all other assays, including virulence in *G. mellonella* (Figures 5–7). The murine pneumonia model was chosen because lung infections are among the most common *A. baumannii* infections, and AB5075 virulence has been characterized in this model (Jacobs et al., 2014a). As shown in Figure 8, the wild-type strain killed 85% of mice by 7 days post-infection. However, the *cusR* mutant strain was attenuated in this model; death was delayed relative to wild-type and only 65% of the mice died. Virulence of the *cusR* mutant strain was restored by complementation of the *cusR* gene in trans; 85% of the mice were killed by the complemented strain by 7 days post-infection. The *copD* mutant strain showed an even more dramatic virulence defect and only killed 10% of mice as compared to 90 and 60% by the wild-type and complemented strains, respectively. The ability to functionally complement both the *copD* and *cusR* mutant strain phenotypes in the pneumonia model further supports our conclusions concerning failure to achieve complementation in the *G. mellonella* model. In summary, both CusR and CopD individually contribute to virulence of *A. baumannii* in the murine pneumonia model.

## Discussion

Novel therapeutics are desperately needed to treat infections with drug-resistant *A. baumannii* and other superbugs. Because copper homeostasis is an essential process and copper ions themselves have potent toxicity, we sought to identify important copper resistance determinants in *A. baumannii*; we theorize that these could potentially be targeted with future therapeutics. Using a focused approach, we identified genes in *A. baumannii* with putative roles in copper resistance, and we demonstrated that 8 of 21 tested genes individually contributed to *in vitro* copper resistance of AB5075; mutant strains displayed significant copper sensitive phenotypes during planktonic growth (Figures 2, 3). Mutation of genes carried in region D caused the largest increase in copper sensitivity, indicating that this region is crucial for high levels of resistance. Furthermore, when a plasmid-borne copy of region D was introduced into *A. baumannii* strains that naturally lacked region D, copper resistance was dramatically increased (Figure 4). Moreover, strains bearing mutations of genes in region D showed enhanced copper-dependent killing even when found in a biofilm structure (Figure 5). The copper sensitivity is likely due to the fact that many of the mutant strains (*copB, cueO*, *cusR*, *cusS, copD*) demonstrated markedly increased accumulation of intracellular copper as compared to wild-type AB5075, which was able to efficiently efflux copper ions (Figure 6). Finally, when tested *in vivo* in *G. mellonella* and a murine pneumonia model, the copper sensitive mutant strains showed distinct attenuation in virulence. In the *G. mellonella* model, the mutant strains killed less *G. mellonella* than the wild-type strain, with the strongest attenuation observed with the *pcoB*, *cueO*, *cusS*, and *copD* mutant strains (Figure 7). Similarly, in the murine mouse model, the *cusR* and *copD* mutant strains were also attenuated and the attenuation phenotype was able to be complemented (Figure 8). *En masse*, our data indicate that copper resistance is mediated by many genes in AB5075 and show that copper resistance contributes to virulence.

**FIGURE 9:**
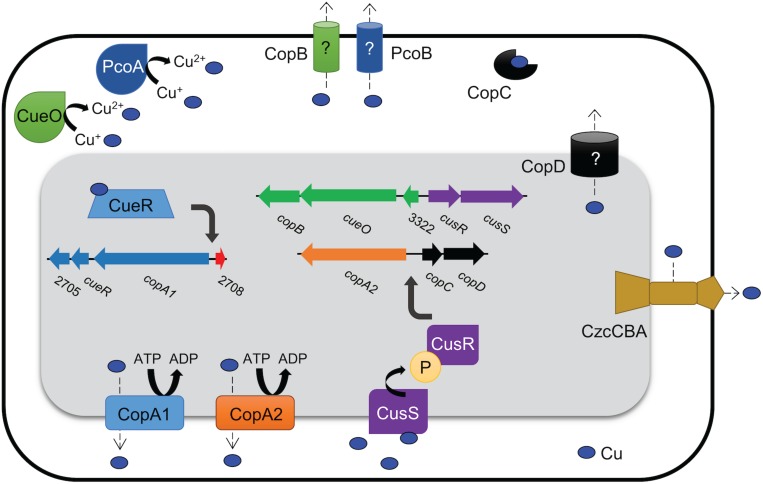
Model of putative copper resistance mechanisms in AB5075. Predicted protein identifications were determined by amino acid sequence homology to known proteins from other bacteria, and proteins with putative copper resistance functions are indicated. In the mutational analysis described herein, proteins putatively involved in transport (PcoB, CopB, CopD), oxidation (PcoA, CueO), chaperoning (CopC), and regulation (CusR, CusS) individually contributed to copper resistance in AB5075. Copper ATPases are highly conserved inner-membrane proteins that efflux copper ions out of the cytoplasm; AB5075 appears to have two copper ATPases: CopA1 and CopA2. The RND family efflux pump CzcCBA may efflux copper from the periplasm to the extracellular milieu. Three other putative membrane proteins in AB5075 may also have roles in copper transport, though the function of these proteins is not yet well-defined in any bacteria (CopB, PcoB, and CopD). Periplasmic copper oxidases reduce damage by converting Cu^+^ to the less toxic Cu^2+^; CueO and PcoA are both predicted copper oxidases. CopC is a highly conserved copper chaperone that is found in the periplasm; it prevents damage by binding and sequestering copper ions and may facilitate movement of copper ions between other copper-related proteins, i.e., delivering copper ions to an efflux pump. Two well-conserved putative regulatory systems are also present in AB5075: CueR senses cytoplasmic copper levels and then upregulates copper resistance genes in its copper-bound form, and CusRS is a two-component system that is thought to be activated by periplasmic copper. The regulons of CueR and CusR have not yet been defined in AB5075, though putative binding sites have been identified in regions B and D, respectively, and these putative target genes are indicated in the figure.

Though the absolute functions of the proteins encoded by each of the genes showing homology to copper related systems remains to be determined, based on predicted functions, a model for the mechanisms of copper resistance in AB5075 was created (Figure 9). When thinking about this model, it is important to keep in mind that based on the mutational analysis, proteins putatively involved in transport (PcoB, CopB, CopD), oxidation (PcoA, CueO), chaperoning (CopC), and regulation (CusR, CusS) individually contributed to copper resistance in AB5075 in our tested *in vitro* conditions. However, this does not negate a possible role for the other predicted copper-related factors within other environments. Also, it should be noted that the model proposed by another group for copper resistance proteins in AB5075 differs slightly from ours (Alquethamy et al., 2019). Furthermore, our model does not include any potential interactions with other biologically relevant heavy metals. In copper-related systems that have been studied in other bacteria, it has been shown that there is sometimes interaction with other heavy metals, e.g., silver (Rensing et al., 2000; Gudipaty et al., 2012); therefore, a limitation of our study is that we only investigated the role of these genes in copper resistance. Thus, there is potential that these genes could also contribute to resistance to other metals; such possible roles warrant further study.

We utilized multiple assays to analyze *in vitro* copper resistance and to assess virulence. In many cases, we observed corroborating results across multiple assays. For instance, we observed similar patterns of sensitivity in our planktonic and biofilm growth assays. The four strains with the greatest increase in copper sensitivity during planktonic growth (*cusR, cusS, copC, copD*) also demonstrated sustained, increased copper sensitivity in the context of biofilm growth. The four additional strains that showed more moderate increases in copper sensitivity in the planktonic growth assay, displayed temporarily increased (*copB*, *cueO*) or no change in sensitivity (*pcoA*, *pcoB*) relative to the wild-type background in the context of biofilm growth. Interestingly, relative to the most copper sensitive mutant strains, the *copB* and *cueO* mutant strains seemed to be more copper sensitive in the biofilm assay than in the planktonic growth assay; the reason for this change in the pattern of sensitivity among the strains is unknown, however, different culture conditions (rich vs. minimal media) could play a role. Additionally, we did not observe a direct correlation between *in vitro* copper sensitivity and attenuation of virulence in the *G. mellonella* infection model. For example, the two most attenuated mutant strains in the *G. mellonella* model (*pcoB* and *cueO*) had only moderate copper sensitivity in the planktonic growth and biofilm survival assays, while the *cusR* and *copC* mutant strains demonstrated strong phenotypes in *in vitro* assays but were only mildly attenuated for virulence in *G. mellonella*. These differences among assays highlight the importance of the methods chosen to assess genes of interest; the roles of individual genes may vary depending on environmental conditions, and this will affect the experimental outcomes and the conclusions drawn. Indeed, experimental differences may account for contrasting results in this work and those published recently by another group (Alquethamy et al., 2019). Under the conditions we tested, we did not observe increased copper sensitivity of either of the copper ATPase mutant strains (Supplementary Figure S1) or attenuation in the *G. mellonella* infection model (Supplementary Figure S5). We originally hypothesized that perhaps CopA1 and CopA2 were functionally redundant, resulting in an inability to observe a copper-sensitive phenotype. However, a recently published study of the copper ATPases in AB5075 found that the gene referred to as *copA1* in our publication (ABUW_2707, region B), but not *copA2* (ABUW_3325, region D), contributed to copper resistance (Alquethamy et al., 2019); those results suggest that the CopA1 and CopA2 proteins are not functionally redundant. While the exact reason for the difference in study results is not clear, it is worth noting that the experimental methods used were different, e.g., growth medium (which dramatically affects free copper concentrations) and infection model; additionally, the various utilized mutant strains differed in their site of transposon insertion and transposon orientation, which may cause differential polar effects. Given the disparity in the results of the studies, it is currently unclear what the individual roles of these copper ATPases are in *A. baumannii*, and additional studies will be required to clearly define the role of these factors in copper resistance.

Our studies identified many copper resistance proteins that individually contributed to virulence in the chosen models; these proteins have a variety of putative functions including copper efflux, oxidation, chaperoning, and regulation. Copper-related genes with similar functions have been shown to be important for virulence in many bacterial and fungal pathogens including *Pseudomonas aeruginosa*, *Pseudomonas fluorescens, Xanthomonas campestris* pv. campestris, *Xanthomonas citri* subsp. *citri*, *Streptococcus pneumoniae*, *Streptococcus mutans*, *Salmonella* Typhimurium, *Mycobacterium tuberculosis*, *Neisseria gonorrhoeae*, *Listeria monocytogenes*, *E. coli*, *Staphylococcus aureus*, *Campylobacter jejuni*, *Aspergillus fumigatus*, and *A. baumannii* (Francis and Thomas, 1997; Schwan et al., 2005; Zhang and Rainey, 2007; Hu et al., 2009; White et al., 2009; Achard et al., 2010; Gonzalez-Guerrero et al., 2010; Osman et al., 2010; Ward et al., 2010; Corbett et al., 2011; Hsiao et al., 2011; Shafeeq et al., 2011; Wolschendorf et al., 2011; Yan and Wang, 2011; Djoko et al., 2012; Marrero et al., 2012; Chaturvedi et al., 2014; Shi et al., 2014; Subashchandrabose et al., 2014; Johnson et al., 2015; Garcia et al., 2016; Park et al., 2017; Wiemann et al., 2017; Gardner and Olson, 2018; Purves et al., 2018; Zapotoczna et al., 2018; Alquethamy et al., 2019). Of note, three types of copper-related proteins that have been previously shown to contribute to virulence of other bacteria were found to also contribute to virulence of *A. baumannii*: copper ATPases, copper oxidases, and copper chaperones. In the majority of the previous studies, the mutated copper resistance gene of interest was a copper ATPase, homologous to *copA1* and *copA2* of AB5075. Of note, a role for CopA1 in virulence of *A. baumannii* was recently described (Alquethamy et al., 2019). However, we did not observe copper sensitivity or reduced virulence in either of our copper ATPase mutant strains (Supplementary Figures S1, S5). Copper oxidases have been shown to be important for virulence of *S. pneumoniae*, *C. jejuni*, and *S. aureus* (Achard et al., 2010; Gardner and Olson, 2018; Zapotoczna et al., 2018). Here, we demonstrated that both copper oxidases of AB5075, PcoA and CueO, contribute to virulence of *A. baumannii* as well. Similarly, chaperone proteins have been previously linked to virulence in *S. pneumoniae* and *S. mutans* (Johnson et al., 2015; Garcia et al., 2016), and we found that the putative chaperone CopC contributes in *A. baumannii*. Though their functions may be similar, it is unclear how related CopC of *A. baumannii* is to the chaperones of other bacteria, as these chaperone proteins are small and have minimal sequence identity or similarity. While these three types of proteins are interesting as putative drug targets, an important consideration in identifying novel drug targets is assessment of homology with host proteins and potential toxicity from off-target activity. Copper ATPase and oxidase proteins have amino acid homology to human proteins, and the copper ATPases are also functionally similar. Thus, designing therapeutics that target these functions may be complicated by homology to the essential human proteins, e.g., human copper ATPases ATP7A and ATP7B. While not insurmountable, homology to host proteins certainly makes design of novel therapeutics more challenging.

Of particular interest, we identified a number of copper-associated proteins that have not previously been shown to contribute to virulence in other bacterial species. To our knowledge, this is the first publication to report attenuated virulence in any bacterial species of strains lacking the putative transporters PcoB, CopB, or CopD (Wolschendorf et al., 2011; Subashchandrabose et al., 2014). We also observed attenuated virulence of the strains bearing mutations in either portion of the two-component system CusRS. To our knowledge, this is the first report that this copper-sensing two-component system contributes to virulence of a bacterial species during mammalian infection. Additionally, none of these proteins share amino acid sequence homology with human proteins, and therefore may be targeted more readily.

While the focus of this work was copper resistance in *A. baumannii*, we note that copper homeostasis mechanisms are well-conserved across bacterial species; the open reading frames studied here were originally identified in AB5075 due to their conserved predicted protein sequence. Therefore, we expect that any novel therapeutics that are targeted against these proteins may have broad-spectrum antibacterial activity. Despite the fact that individual proteins are well-conserved, the presence of these proteins or their homologs varies across bacterial species and even amongst strains (Hernandez-Montes et al., 2012). Indeed, we observed that not all the same copper resistance proteins are present in all clinical isolates of *A. baumannii*. Thus, these differences will be an important consideration when choosing which copper resistance genes may be the best therapeutic targets. Given all of these possible targets, copper homeostasis components could provide an attractive starting place for the development of future antimicrobial therapies.

The immune system has harnessed the antimicrobial power of copper for use against pathogens and copper-mediated immune mechanisms are known to play a role in clearance of bacterial pathogens (Sheldon and Skaar, 2019). Because the innate immune response of phagocytic cells is crucial for clearance of *A. baumannii* infection, we hypothesize that copper resistance contributes to virulence of this pathogen by enhancing immune evasion and survival *in vivo*. Therefore, we predict that novel therapeutics targeting copper resistance of bacterial pathogens would enhance immune clearance by reducing bacterial survival in the copper-rich phagosomal compartment. The role of copper in the immune system has been demonstrated by prior studies that utilized copper-replete and copper-deficient cell lines and animal models. For example, macrophages that are pre-incubated with copper are able to kill more internalized bacteria than controls (White et al., 2009). Conversely, copper-deficient macrophages or macrophages that are unable to mobilize copper to the phagosomal compartment due to loss of function of ATP7A are less efficient at killing bacterial pathogens (White et al., 2009; Achard et al., 2012; Ladomersky et al., 2017). The importance of copper in immunity has also been demonstrated on an organismal level. Animals with copper-deficient diets are more susceptible to infection, while those with copper-rich diets more efficiently clear infection (Newberne et al., 1968; Wolschendorf et al., 2011; Gardner and Olson, 2018). Furthermore, mice with a myeloid-specific knockout of ATP7A, the copper ATPase that pumps toxic copper ions into the macrophage phagosome, are more susceptible to infection with *Salmonella* Typhimurium (Ladomersky et al., 2017). Comparable future studies using *A. baumannii* will help to shed light on whether copper resistance of this organism aids in virulence by enabling evasion of host copper-mediated immune strategies.

In summary, we identified several additional copper resistance proteins that may serve as potential therapeutic targets, including copper transporters PcoB, CopB, and CopD, chaperone CopC, oxidases CueO and PcoA, and regulatory proteins CusR and CusS. Future work using targeted antibodies and small molecules will seek to determine the efficacy of targeting these copper resistance proteins as a successful treatment strategy. Given the relative conservation of these proteins and pathways among bacteria, we believe several are viable therapeutic targets to treat *A. baumannii* infection as well as other multidrug-resistant bacterial pathogens, for which novel treatments are urgently needed.

## Data Availability Statement

The datasets generated for this study are available on request from the corresponding author.

## Ethics Statement

The animal study was reviewed and approved by the Institutional Animal Care and Use Committee at the Walter Reed Army Institute of Research.

## Author Contributions

CW, SM, DZ, and DM conceived and designed the experiments. CW, HN, YA, RR, AJ, SS, and RA-T performed the experiments. CW and DM wrote the manuscript.

## Disclaimer

Material has been reviewed by the Walter Reed Army Institute of Research (WRAIR). There is no objection to its presentation and/or publication. The opinions and assertions contained herein are the private ones of the authors and are not to be construed as official or reflecting the views of the Department of Defense, the Uniformed Services University of the Health Sciences, the Department of the Army, or any other agency of the U.S. Government. Research was conducted in an AAALACi-accredited facility in compliance with the Animal Welfare Act and other federal statutes and regulations relating to animals and experiments involving animals and adheres to principles stated in the Guide for the Care and Use of Laboratory Animals, NRC Publication, 2011 edition.

## Conflict of Interest

The authors declare that the research was conducted in the absence of any commercial or financial relationships that could be construed as a potential conflict of interest.
