# Supplementary material for: Characterization of Acinetobacter baumannii Copper Resistance Reveals a Role in Virulence
Source: Front Microbiol. 2020 Feb 6;11:16. doi: 10.3389/fmicb.2020.00016 (PMC7015863; doi:10.3389/fmicb.2020.00016)
Supplement: Supplementary file 1 [file Data_Sheet_1.pdf]

Supplementary Material

1.1 Supplementary Figures

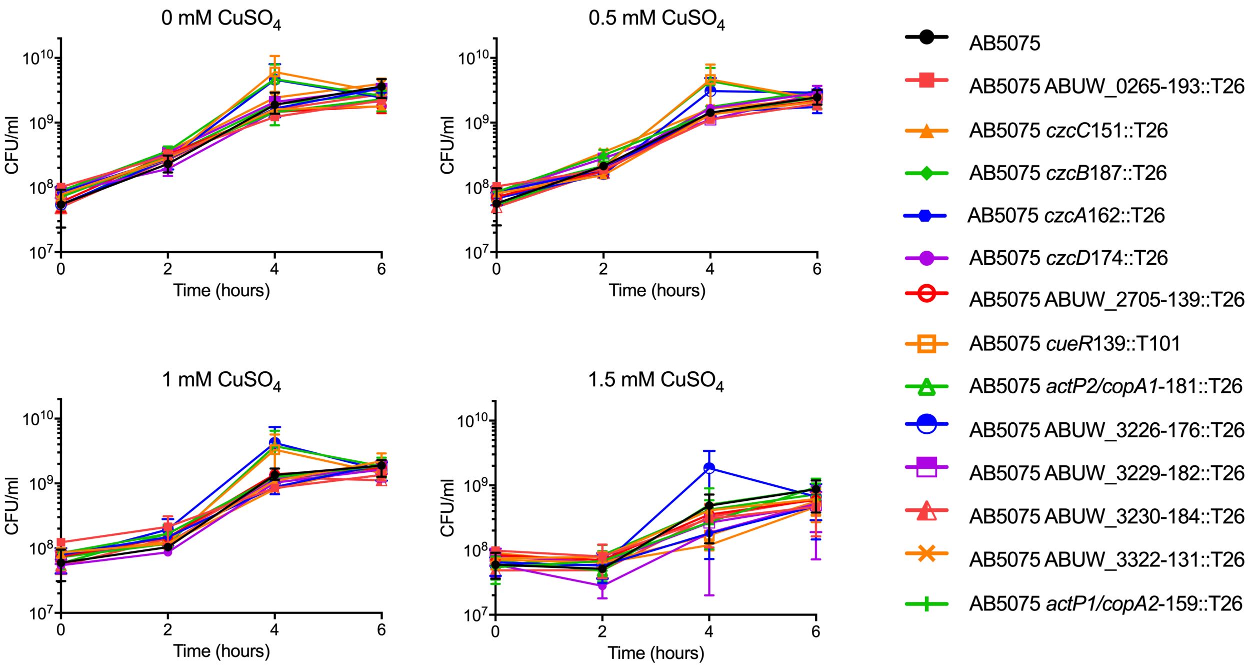

Supplementary Figure 1. Growth of AB5075 mutant strains that displayed no change in copper sensitivity. Strains were grown for 6 hours at 37ºC in M9 medium supplemented with the concentration of $CuSO_4$ indicated in the legend. Growth was measured both by turbidity (OD_{600}, data not shown) and enumeration of viable colonies (CFU/mL). Two biologically independent experiments were completed, and the data are grouped by copper concentration and are presented as mean and range.

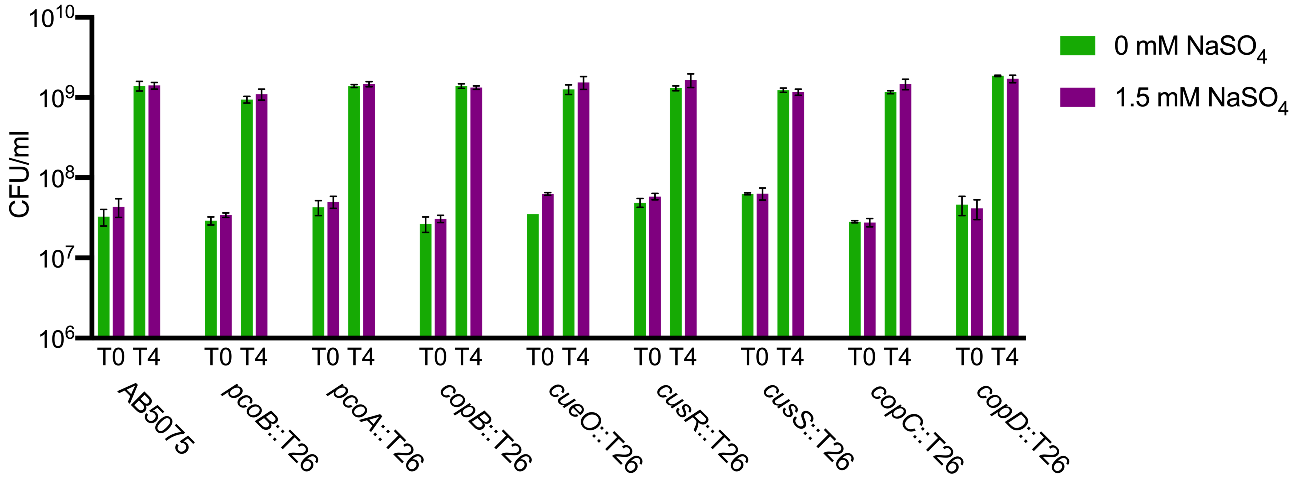

Supplementary Figure 2. Growth of AB5075 mutant strains in sodium sulfate. Strains were grown for 4 hours at 37ºC in M9 medium with or without an additional 1.5 mM $NaSO_4$. Growth was measured by enumeration of viable colonies (CFU/mL). The data are presented as the geometric mean and SEM of three biologically independent experiments. Two-way ANOVA with Tukey's adjustment for multiple comparisons was used to compare growth in each sulfate concentration at each timepoint; no comparisons were statistically significant ($P > 0.05$).

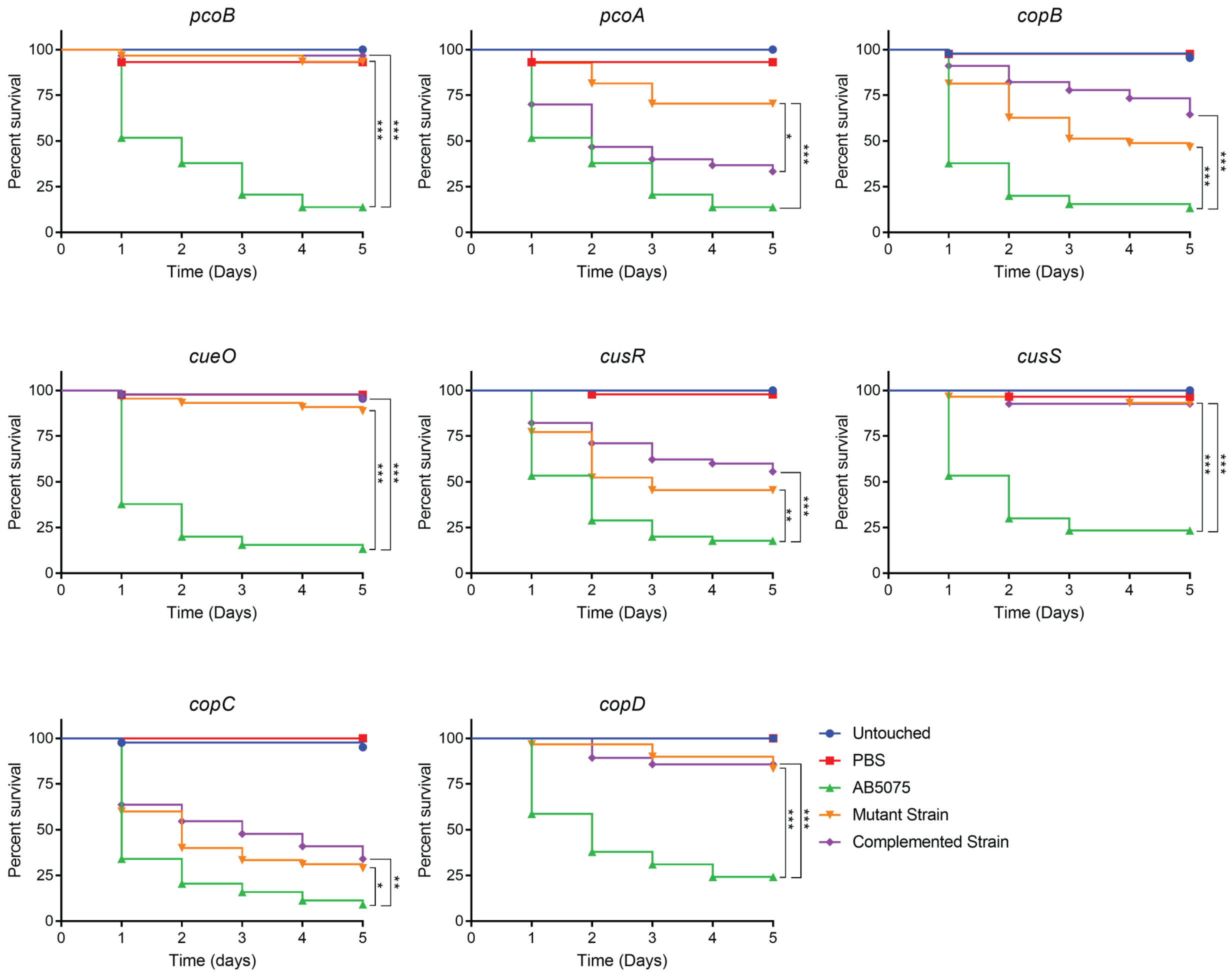

Figure S3. Survival of *Galleria* infected with AB5075 mutant and complemented strains. *Galleria* were injected with approximately 5.0 x 10^4 CFU of the indicated strain and survival was monitored for 5 days. The gene interrupted in each mutant strain and restored in the complemented derivative is indicated in the graph title. Experiments were repeated using two or three different orders of larvae with 12-15 larvae per experimental group; the total n ranged from 25 to 45 larvae per strain. Kaplan-Meier survival curves were compared (excluding Untouched and PBS controls) using the Mantel-Cox test with Holm's correction for multiple comparisons. Statistically significant comparisons (mutant strain vs. wild-type strain, mutant strain vs. complemented derivative, complemented derivative vs. wild-type strain) are indicated by bars with asterisks signifying the *P* value as follows: *, $P < 0.05$; **, $P < 0.01$; ***, $P < 0.001$.

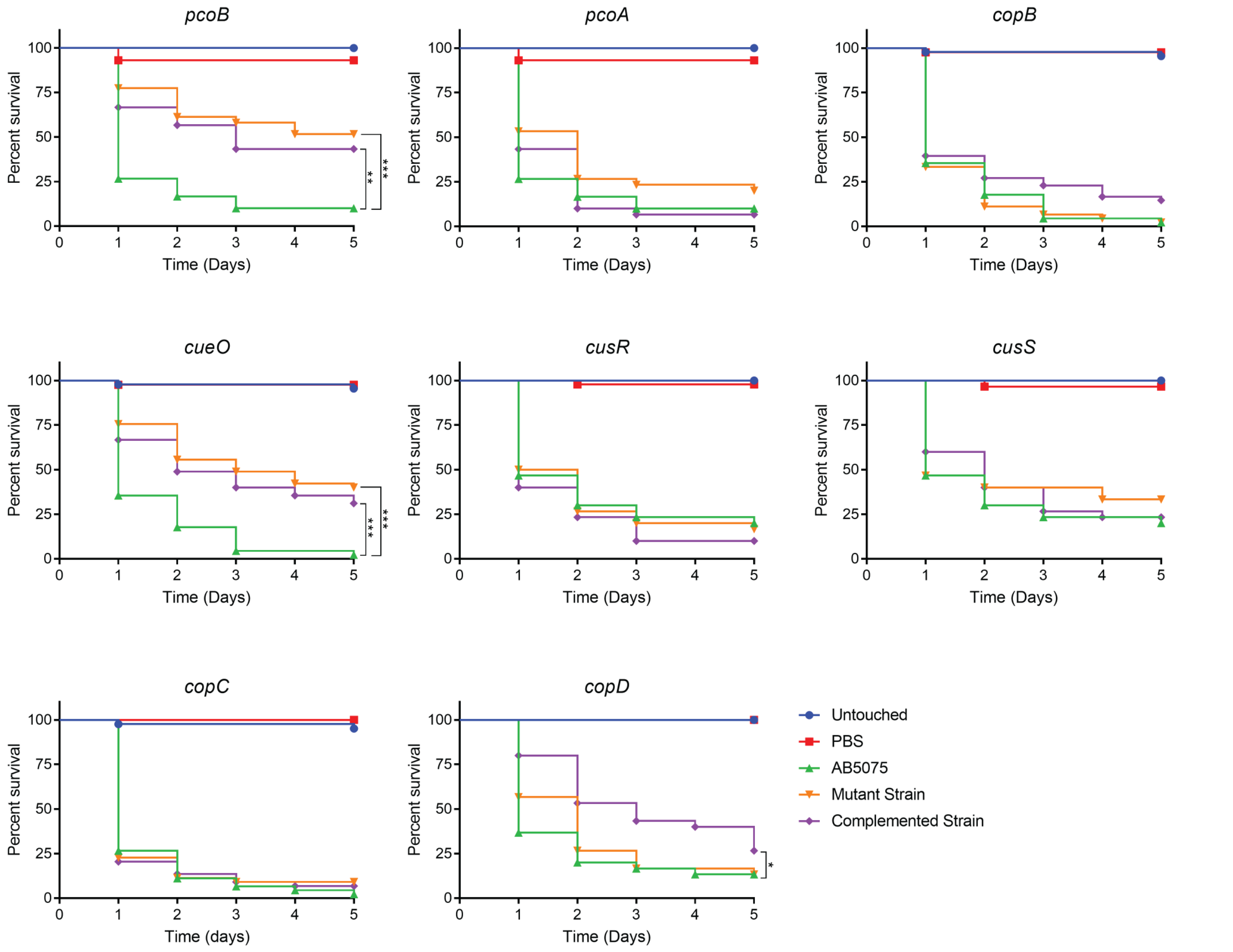

Figure S4. Survival of *Galleria* infected with a high dose of AB5075 mutant and complemented strains. *Galleria* were injected with approximately 5.0 x 10^5 CFU of the indicated strain and survival was monitored for 5 days. The gene interrupted in each mutant strain and restored in the complemented derivative is indicated in the graph title. Experiments were repeated using two different orders of larvae with 12-18 larvae per experimental group; the total n ranged from 25 to 48 larvae per strain. Kaplan-Meier survival curves were compared (excluding Untouched and PBS controls) using the Mantel-Cox test with Holm's correction for multiple comparisons. Statistically significant comparisons (mutant strain vs. wild-type strain, complemented derivative vs. wild-type strain) are indicated by bars with asterisks signifying the *P* value as follows: **, $P < 0.01$; ***, $P < 0.001$.

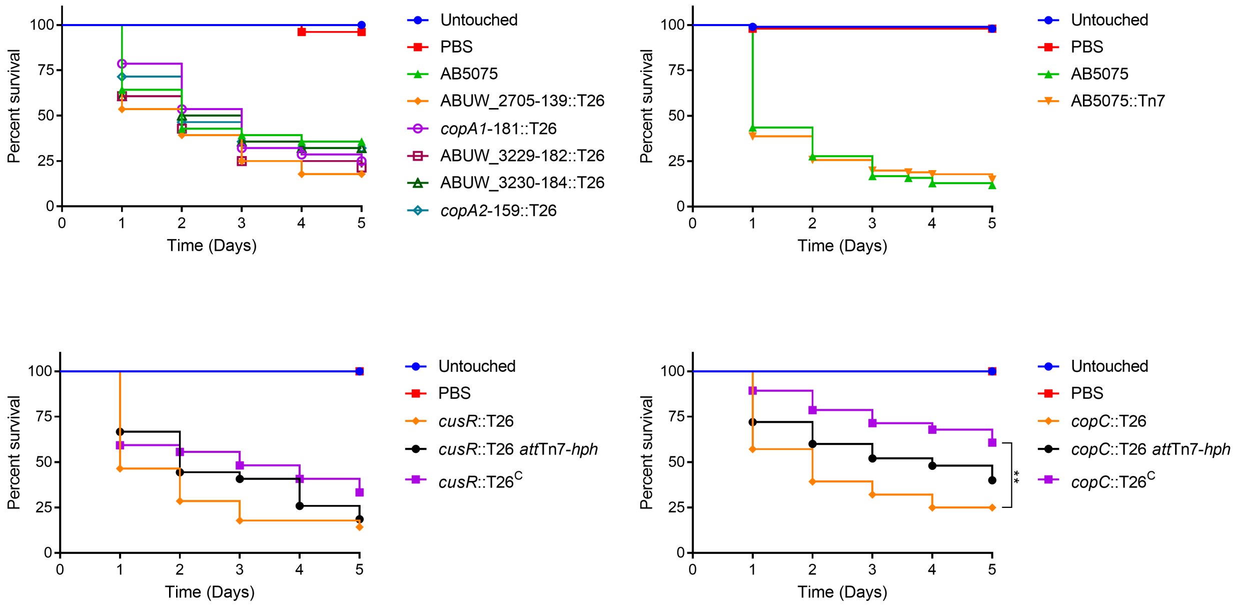

Figure S5. Survival of *Galleria* infected with additional mutant strains and Tn*7*-containing strains. *Galleria* were injected with approximately 1.0 x 10^5 CFU (actual doses ranged from 6.75 x 10^4 - 1.40 x 10^5 CFU) of the indicated strain and survival was monitored for 5 days. Kaplan-Meier survival curves were compared (excluding Untouched and PBS controls) using the Mantel-Cox test with Holm's correction for multiple comparisons. For the additional mutant strains (top left), two biologically independent experiments were conducted using two separate batches of caterpillars; the combined data are presented with 26-28 caterpillars per group. No strains were significantly different from the wild-type. For the comparison of wild-type AB5075 to AB5075::Tn*7* (top right), six biologically independent experiments were conducted using six separate batches of caterpillars; the combined data are presented, with 96-101 total caterpillars per group. The strains were not significantly different. For the strains derived from the *cusR* mutant strain (lower left) and *copC* mutant strain (lower right), two biologically independent experiments were conducted using two separate batches of caterpillars; the combined data are presented with 25-28 caterpillars total per group. Statistically significant comparisons (mutant strain vs. complemented derivative) are indicated by bars with asterisks signifying the *P* value: **, $P < 0.01$.
